# New cladotherian mammal from southern Chile and the evolution of mesungulatid meridiolestidans at the dusk of the Mesozoic era

**DOI:** 10.1038/s41598-021-87245-4

**Published:** 2021-04-07

**Authors:** Agustín G. Martinelli, Sergio Soto-Acuña, Francisco J. Goin, Jonatan Kaluza, J. Enrique Bostelmann, Pedro H. M. Fonseca, Marcelo A. Reguero, Marcelo Leppe, Alexander O. Vargas

**Affiliations:** 1 CONICET-Sección Paleontología de Vertebrados, Museo Argentino de Ciencias Naturales “Bernardino Rivadavia”, Av. Ángel Gallardo 470, C1405DJR CABA, Argentina; 2 Red Paleontológica U-Chile, Laboratorio de Ontogenia y Filogenia, Departamento de Biología, Facultad de Ciencias, Universidad de Chile, Las Palmeras 3425, 7750000 Ñuñoa, Santiago Chile; 3 KayTreng Consultores SpA, José Domingo Cañas 1640, Apt. 1502, 7750000 Ñuñoa, Santiago Chile; 4 CONICET-División Paleontología Vertebrados, Museo de La Plata, Paseo del Bosque s/n, B1900FWA La Plata, Argentina; 5 Fundación de Historia Natural Félix de Azara, Universidad Maimónides, Hidalgo 775, C1405BCK CABA, Argentina; 6 Instituto de Ciencias de la Tierra, Facultad de Ciencias, Universidad Austral de Chile, Los Laureles s/n, 5090000 Valdivia, Chile; 7 Programa de Doctorado en Ciencias Mención Ecología y Evolución, Universidad Austral de Chile, Los Laureles s/n, 5090000 Valdivia, Chile; 8 Museo Regional de Aysén, Kilómetro 3 camino a Coyhaique Alto, Coyhaique, Región de Aysén Chile; 9 Programa de Pós-Graduação em Geociências, Instituto de Geociências, Universidade Federal do Rio Grande do Sul, Av. Bento Gonçalves, 9500 Agronomia, Porto Alegre, RS 91501-970 Brazil; 10 Laboratorio de Paleobiología de Antártica y Patagonia, Instituto Antártico Chileno, Plaza Muñoz Gamero 1055, 6200000 Punta Arenas, Chile

**Keywords:** Evolution, Palaeontology

## Abstract

In the last decades, several discoveries have uncovered the complexity of mammalian evolution during the Mesozoic Era, including important Gondwanan lineages: the australosphenidans, gondwanatherians, and meridiolestidans (Dryolestoidea). Most often, their presence and diversity is documented by isolated teeth and jaws. Here, we describe a new meridiolestidan mammal, *Orretherium tzen* gen. et sp. nov., from the Late Cretaceous of southern Chile, based on a partial jaw with five cheek teeth *in locis* and an isolated upper premolar. Phylogenetic analysis places *Orretherium* as the earliest divergence within Mesungulatidae, before other forms such as the Late Cretaceous *Mesungulatum* and *Coloniatherium*, and the early Paleocene *Peligrotherium*. The *in loco* tooth sequence (last two premolars and three molars) is the first recovered for a Cretaceous taxon in this family and suggests that reconstructed tooth sequences for other Mesozoic mesungulatids may include more than one species. Tooth eruption and replacement show that molar eruption in mesungulatids is heterochronically delayed with regard to basal dryolestoids, with therian-like simultaneous eruption of the last premolar and last molar. Meridiolestidans seem endemic to Patagonia, but given their diversity and abundance, and the similarity of vertebrate faunas in other regions of Gondwana, they may yet be discovered in other continents.

## Introduction

Before the establishment of metatherians and eutherians as the dominant mammals of the Cenozoic terrestrial ecosystems of South America^1–3^, two main non-tribosphenic mammalian clades achieved large diversity and dominance during the Late Cretaceous: the gondwanatherian allotherians^4–7^ and the meridiolestidan cladotherians^8–16^. Gondwanatherians achieved a Gondwanan distribution, with a dozen species as well as indeterminate records from the Upper Cretaceous of Tanzania, Argentina, Chile, Madagascar, and India, as well as the Paleogene of Argentina, Antarctica and perhaps Peru. They had a specialized dentition for herbivorous feeding habits, including gliriform incisors and hypsodont molariforms in some taxa^7,17^. In contrast, meridiolestidans likely represent an endemic group of South American dryolestoid cladotherians with a typically reversed triangle pattern for the cheek teeth, though lacking the tribosphenic design (i.e., absence of a protocone and a basined talonid)^18,19^.

**Figure 1:**
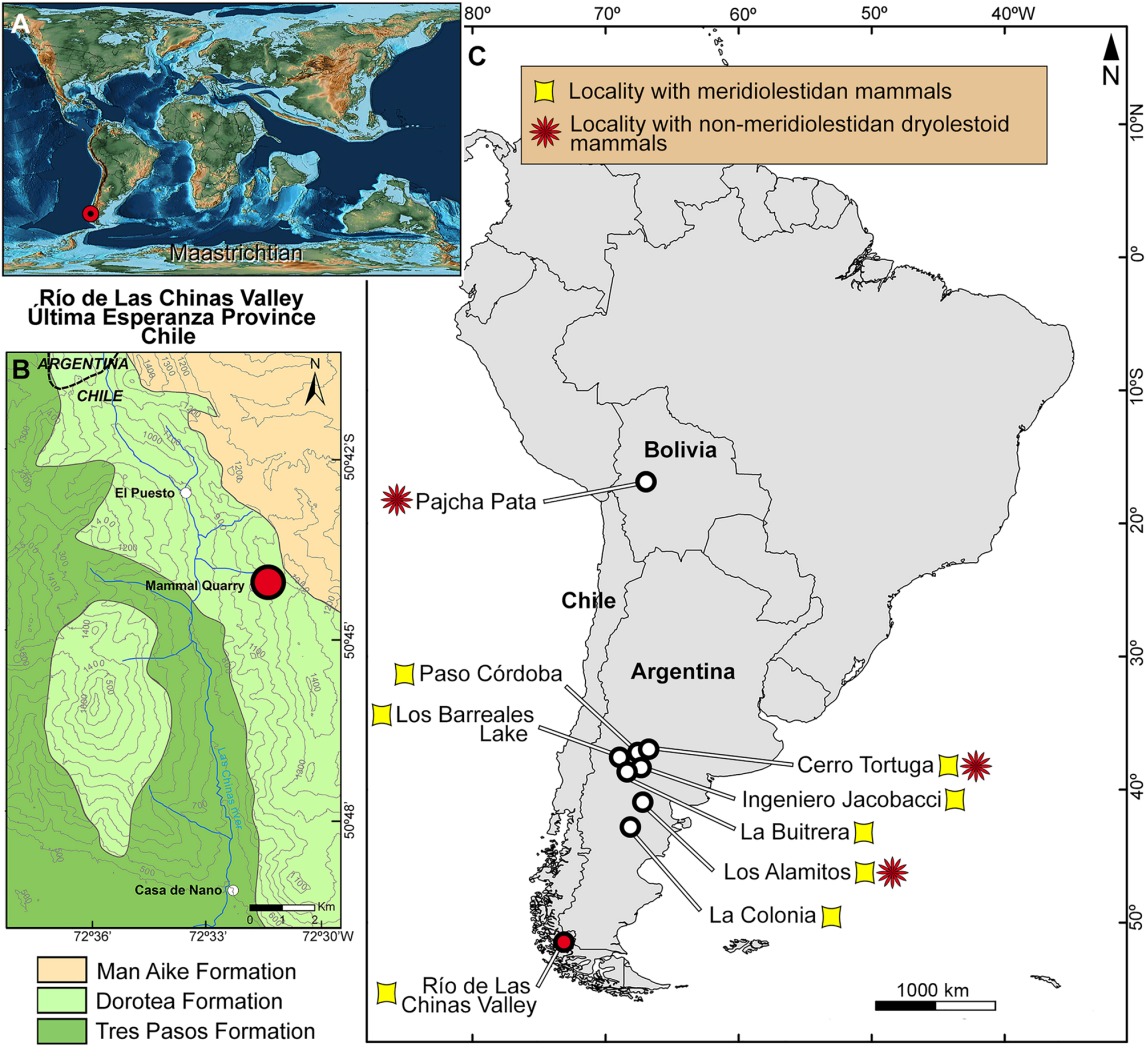
Location map of Río de Las Chinas Valley, Estancia Cerro Guido, Última Esperanza Province, Chilean Patagonia. (**A**) Mammal Quarry location during the Late Cretaceous. Map modified from Scotese^85^. (**B**) Mammal Quarry at the Río de Las Chinas Valley, with exposed geological formations. (**C**) Localities with osteological records of Cretaceous dryolestoid mammals from South America.

The fossil record of meridiolestidans includes badly preserved specimens that do not allow a species-level determination^20,21^, species of still poorly understood affinities (e.g., *Casamiquelia rionegrina*, see review in Rougier et al.^15^), and species that are clustered into two main branches: the non-bunodont *Cronopio dentiacutus*, *Leonardus cuspidatus*, *Necrolestes patagonensis*, and *N. mirabilis*, and the bunodont mesungulatoids *Reigitherium bunodontum*, *Mesungulatum houssayi*, *M. lamarquensis*, *Paraungulatum rectangularis*, *Coloniatherium cilinskii*, and *Peligrotherium tropicalis*^8–15,22^ (Fig. 1). Mesungulatids (the above species, but likely excluding *Reigitherium*; see our phylogenetic analysis below) have bunodont postcanines, molarization of the last premolars, and labio-lingually extended mesial and distal cingula on their upper and lower cheek teeth^8,10,12,13,16,23,24^. Meridiolestidans were at the core of mammalian radiations in South America after the Late Cretaceous event known as Cretaceous Terrestrial Revolution^25^, with their molar morphology suggesting a trend towards greater ecological diversity^15^. Meridiolestidans survived the mass extinction event at the end of the Mesozoic Era and persisted as a vestigial group in the Cenozoic, including *Peligrotherium tropicalis* from the early Paleocene of Patagonia^24,26–28^, a bizarre indeterminate taxon from the Eocene of Antarctica^29^, and two species of *Necrolestes* from the early Miocene of Patagonia^14,30,31^. In addition to gondwanatherians and meridiolestidans, the current record of Late Cretaceous mammals in South America includes two species of dryolestidans from Argentina closely related to Laurasian forms (i.e., *Groebertetherium stipanicici* and *G. allenensis*^11,15^), as well as a handful of other records of uncertain affinities from Peru, Bolivia and Brazil^32–35^.

The dentition of dryolestoid mammals illustrates a radiation of pre-tribosphenic mammals, a group of stem Theria in which metatherians and eutherians are nested (i.e., Theria^36,37^). The composition of Dryolestoidea includes the traditional families Dryolestidae and Paurodontidae^38–42^ (grouped together as Dryolestida), plus the clade Meridiolestida^13,14^. Dryolestidae is the most specious group (~ 20 species)^38^ with almost all of its members coming from Laurasia, from the Middle Jurassic to Early Cretaceous strata from North America and Europe^38–42^ and the Middle Jurassic of Asia^43^. *Groebertherium* spp. from the Late Cretaceous of Patagonia and possibly *Donodon prescriptoris* from the Early Cretaceous of Africa were considered dryolestids or dryolestidans^10,23,42,44^, enlarging the temporal and geographical distribution of the group. Paurodontidae, in its traditional sense, comprises about a dozen species mainly distributed in the Late Jurassic of North America^45,46^, Late Jurassic to Early Cretaceous of Europe^47,48^, and a possible species in the Late Jurassic of Tanzania^38^; however, this family has been considered as paraphyletic in more recent phylogenetic analyses and their members may represent early diverging dryolestidans^49^. The interrelationships of dryolestidans have resulted in disparate topologies according to different phylogenetic studies^13,14,17,22,49^, deserving more in depth analysis. The recognition of meridiolestidans as an endemic group of South American dryolestoids^13^ is much more recent than for these two, mainly Laurasian, traditional clades, which have been known since the late Nineteenth Century^39–41^. The first meridiolestidan recognized was *Mesungulatum houssayi*, which also represents the first undisputed mammalian record for the Mesozoic of South America^50^. Although eutherian affinities were first considered for *Mesungulatum*^50^, an immediate amendment considered it as a pretribosphenic mammal related to dryolestoid cladotherians^8^. By that time, intensive fieldwork led by Dr. José F. Bonaparte resulted in the discovery of a diverse mammal assemblage from the Campanian–Maastrichtian Los Alamitos Formation (northern Patagonia, Argentina), comprising as many as 15 new species of non-therian mammals (excluding gondwanatherians), mostly based upon isolated dental elements. Further materials coming from other localities and ages (e.g., Candeleros, Allen, La Colonia and Salamanca formations) indicated that this diversity was overestimated; in some cases, taxa were recognized on the basis of isolated teeth representing different *loci* of dentition in the same taxon^10–13,15,38,42,51–53^. The foundational stone for the recognition of the South American clade Meridiolestida was based on the large number of mammalian discoveries at the roughly coeval Los Alamitos, Allen and La Colonia formations, plus significant records in the mid-Cretaceous Candeleros Formation and lower Paleocene Salamanca Formation^13^. The whole evidence proves that meridiolestidans evolved more disparate dental and craniomandibular morphotypes than their relatives the dryolestid and paurodontid dryolestoids^8–10,13–15,31^. Contrary to this line of evidence, Averianov et al.^49^ proposed an alternative hypothesis in which meridiolestidans were nested as non-cladotherian trechnotherians related to spalacotheroid “symmetrodontans”. Features used to link meridiolestidans with spalacotheroids are the presence of an anterior lower premolar with well-developed mesial and two distal accessory cusps; the lack of a distinctive talonid; well-developed mesial and distal cingula; mesiodistally compressed roots in lower molars; lack of angular process and a Meckel’s groove in the dentary; and masseteric process (particularly that of *Cronopio*) homologous to the masseteric shelf of some spalacolestines^49^. Wible and Rougier^31^ and Rougier et al.^15^ discussed this hypothesis^49^ and stated that some traits were wrongly scored, such as the absence of an angular process in meridiolestidans (which is present in *Cronopio*, *Peligrotherium* and an unnamed Cretaceous form^13,21,24^) or have a random distribution amongst trechnotherians, not being synapomorphic for both clades (spalacotheroids and meridiolestidans)^15,31^, such as the masseteric process which is only present in *Cronopio* and in few putative “symmetrodontans”^15^. Further, the proposal of meridiolestidans as related to “symmetrodontans”^49^ dismissed several non-dental traits^15,31^. However, given the disparity of craniodental morphologies among meridiolestidans^13–15,31^ and the incompleteness of most taxa, the relationships of this endemic South American group among the trechnotherians are not a fully resolved matter. The sum of cranio-dental evidence provided by the last comprehensive analyses^15,16,22,31^ (including our modified dataset) supports a cladotherian position for meridiolestidans, usually linked to dryolestidans within Dryolestoidea, a scheme we follow here.

Here we describe the southernmost record of a meridiolestidan mammal from the Upper Cretaceous Dorotea Formation at the Magallanes Region of southernmost Chile. Two specimens are referred to a new taxon, *Orretherium tzen* gen. et sp. nov., including an upper last premolar (hypodigm CPAP-5008) and a partial lower jaw with the last two premolars and three molars (holotype CPAP-5007). We discuss its affinities, its biogeographic significance, and the extent of the Mesungulatidae as a distinct family of Meridiolestida. Additionally, the lower premolar-molar series of a single individual considerably improves our understanding on the anatomy and tooth replacement sequence for mesungulatid mammals.

## Results

### Systematic palaeontology

Mammalia Linnaeus 1758^54^.

Meridiolestida Rougier, Apesteguía, and Gaetano 2011^13^.

Mesungulatidae Bonaparte 1986^8^.

*Orretherium tzen* gen. et sp. nov.

(Figs. 2–5, 7).

**Figure 2:**
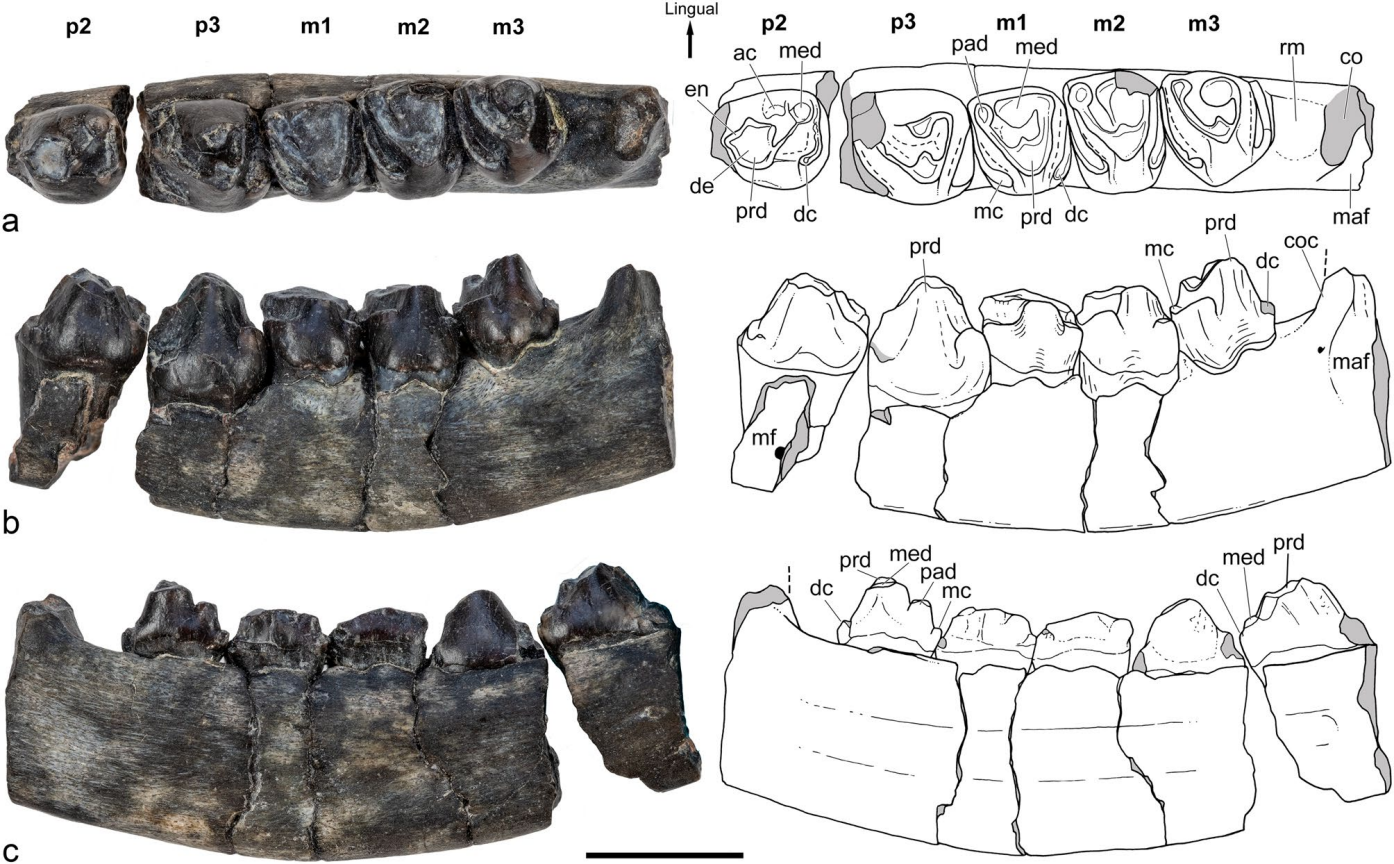
*Orretherium tzen* gen. et sp. nov. (CPAP-5007, holotype). (**a**–**c**) Partial left dentary with p2-p3 and m1-m3 in occlusal (**a**), labial (**b**) and medial (**c**) views. Drawings made by A.G.M. *ac* accessory cusp, *coc* coronoid crest, *dc* distal cingulum, *de* dentine, *en* enamel, *maf* masseteric fossa, *mc* mesial cingulum, *mf* mental foramen, *med* metaconid, *pad* paraconid, *prd* protoconid, *rm* retromolar space. Scale bar: 5 mm.

### Etymology

*Orre* means teeth in the language spoken by the Aonikenk, the original inhabitants of the Patagonian plains in Chile and Argentina, and *therium* from the Greek *thērion*, beast, frequently used for mammals. The species name *tzen* is Aonikenk for five, the number of teeth preserved in the *in locis* sequence.

### Holotype

**Figure 3:**
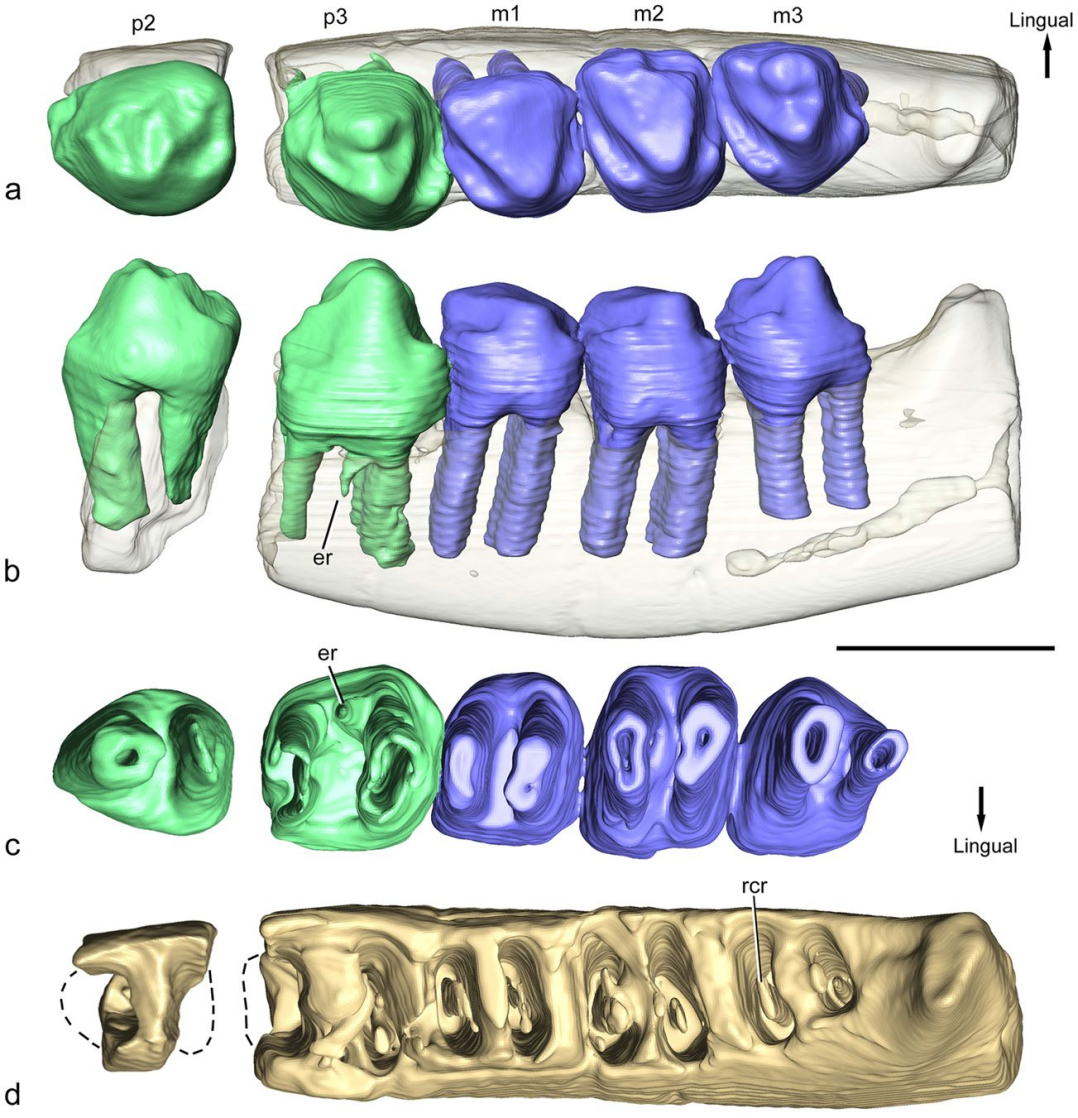
*Orretherium tzen* gen. et sp. nov. (CPAP-5007, holotype). (**a**–**d**) 3D-rendering of transparent partial left dentary with p2-p3 and m1-m3 in occlusal (**a**), labial (**b**) and ventral (**c**) views, highlighting the root morphology, and dentary without teeth in dorsal view (**d**). Images generated by P.H.F.M. using 3D Slicer software. *er* extra root, *rcr* radicular canal of the root. Scale bar: 5 mm.

CPAP-5007, partial left dentary with p2, p3 and m1-m3 (Figs. 2, 3).

### Hypodigm

**Figure 4:**
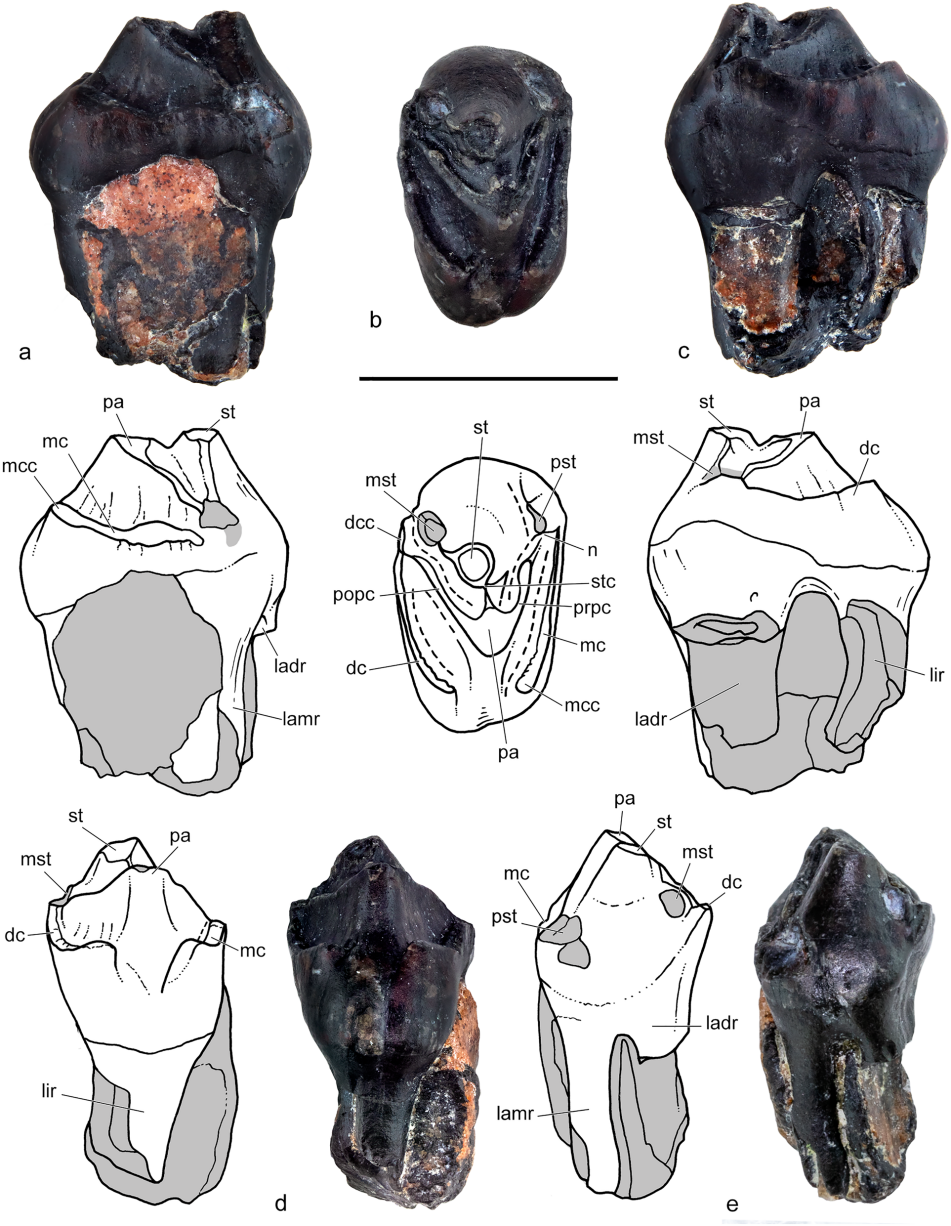
*Orretherium tzen* gen. et sp. nov. (CPAP-5008). (**a**–**e**) last right upper premolar (P3) in mesial (**a**), occlusal (**b**), distal (**c**), lingual (**d**) and labial (**e**) views, with accompanying line drawings. Drawings made by A.G.M. *dc* distal cingulum, *dcc* distal cingulum cusp, *ladr* labiodistal root, *lamr* labiomesial root, *lir* lingual root, *mc* mesial cingulum, *mcc* mesial cingular cusp, *mst* metastyle, *n* notch, *pa* paracone, *pst* parastyle, *popc* postparacrista; prpc, preparacrista; st, stylocone; stc, styloconar crest. Scale bar: 5 mm.

**Figure 5:**
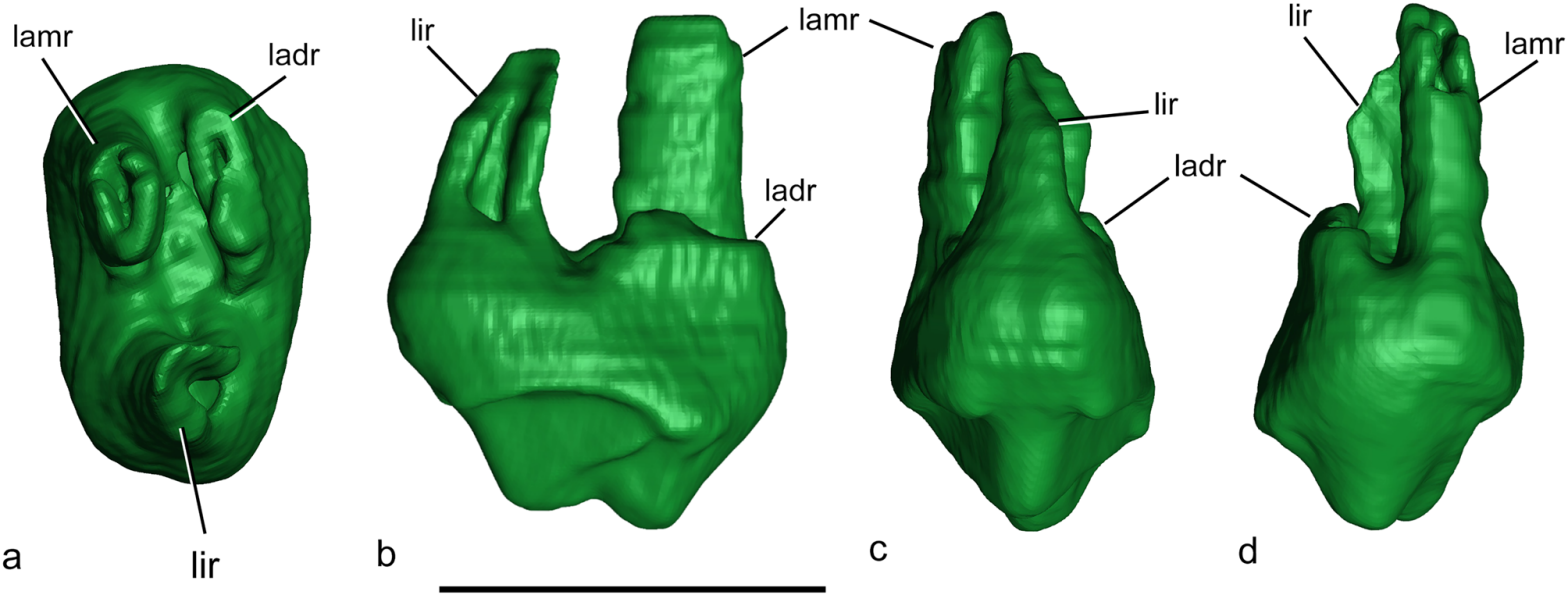
*Orretherium tzen* gen. et sp. nov. (CPAP-5008). (**a**–**d**) 3D rendering of last right upper premolar (P3) in ventral (**a**), distal (**b**), lingual (**c**), and labial (**d**) views. Images generated by P.H.F.M. using 3D Slicer software. *ladr* labiodistal root, *lamr* labiomesial root, *lir* lingual root. Scale bar: 5 mm.

CPAP-5008, almost complete right upper P3 with some remains of the maxillary bone (Figs. 4, 5).

### Comments

CPAP-5008 was found a few meters away from the lower jaw CPAP-5007; even though it cannot be unambiguously referred to the same specimen, it is highly probable taking in account their compatible size, stage of wear, spatial proximity and taphonomic signature in the outcrop (e.g., based on isolated pieces from ~ 10 × 10 m quarry we were able to reconstruct the lower jaw of the holotype, and the outcrop has relatively few mammal individuals/specimens).

### Horizon and locality

All specimens come from a small hill, named Mammal Quarry, located in the Río de Las Chinas valley (50° 42′ S /72° 32′ W), Estancia Cerro Guido, Última Esperanza Province, Magallanes and Chilean Antarctic Region, Chilean Patagonia; lower levels of the Dorotea Formation, late Campanian to early Maastrichtian, Late Cretaceous.

### Diagnosis

Small-sized mesungulatid dryolestoid (sensu phylogenetic hypothesis here obtained and previous ones^13,14,31^) slightly smaller than *Mesungulatum houssayi* and *M. lamarquensis* and larger than the non-mesungulatids *Reigitherium bunodontum* and *Leonardus cuspidatus*. *Orretherium* differs from *Mesungulatum* in having labio-lingually shorter mesial and distal cingula, more developed on the mesiolabial and distolabial sides in m1–m2; mesial and distal cingula mesio-distally longer; more bulbous protoconid; paraconid relatively larger than in *Mesungulatum* and separated of the metaconid by a distinct notch; smaller m1 relative to m2 in *Orretherium* (subequal in *Mesungulatum*). The m3 of *Orreotherium* differs from the inferred m3 of *Mesungulatum* in having a broader and mesially convex mesial cingulum; relatively smaller paraconid and larger metaconid; and more developed swelling at the labial base of protoconid. *Orretherium* differs from *Coloniatherium* in having a relatively smaller p2 compared to p3 and molars; large metaconid in p2, with an accessory lingual cingular cusp next to it; lack of extra roots in p2; wider angle formed by the trigonid cusps in p3; less-developed swelling of the labial base of the protoconid in p3; more labially expanded mesial and distal cingula in p3; postprotocristid projected posterior to the distal surface of the metaconid in p3; only one tiny extra root in p3; m1 smaller than m2 (m1 larger than m2 in the inferred molars of *Coloniatherium*); m1 sub-square instead of sub-rectangular in shape (the latter in *Coloniatherium*). *Orretherium* differs from *Peligrotherium* in having less complex lower cingula in lower premolars and molars; lack of lower labial accessory cuspules in lower molars; more defined main crown cusps (protoconid, paraconid, metaconid) and cristids in p3 and molars; m2 larger than m1 and m3 (in *Peligrotherium* m1 is larger than the remaining molars). *Orretherium* differs from *Reigitherium* in having a triangular configuration of the main cusps of the last premolar and molars; molars with conspicuous paraconid and postprotocristid; labio-lingually narrower molars; lack of labial accessory cuspules in molars. The P3 differs from that of *Coloniatherium* in having a slightly convex mesial wall of the crown; longer post- and preparacristae; metastyle more labially placed; distal cingulum more lingually expanded; slightly concave (instead of convex) lingual wall of the base of the paracone; swelling of the lingual base of the paracone much less reduced. The P3 of *Orretherium* differs from that of *Peligrotherium* in having labio-lingually broader and apico-basally shorter mesial and distal cingula; paracone connected to the stylocone and the metastyle more labially placed to the stylocone. The P3 lacks extra-roots, which are present in *Coloniatherium* and *Peligrotherium*. The P3 differs from the inferred last premolar (P4) of *Reigitherium* in the absence of continuous and clearly differentiated mesial and distal cingula; lack of ectoflexid and extra-labial cusps; and in that the triangular configuration of cusps of the primary trigon is not topologically similar. *Orretherium* shares with other meridiolestidans^13^, while differing from most other dryolestoids, the presence of a large stylocone, similar in size to the paracone, absence of metacone, three lower molars (and inferred three upper molars), a mesiodistally compressed root in the last premolars and molars, and lack of a Meckel’s groove in the inner wall of the dentary.

### Description

#### Lower premolar-molar series

Specimen CPAP-5007 represents the holotype of *Orretherium tzen* and one of the few Late Cretaceous Mesozoic mammals from South America bearing a sequence of five lower cheek teeth *in locis* (Fig. 2). The left lower jaw includes the p2-p3 and m1-m3, identified on the basis of the tooth formula of *Coloniatherium* and *Peligrotherium*^12,15,24^. The crown wear of p3 is less accentuated than that of m1, suggesting that the p3 is a replacement tooth that erupted later than m1 (see below). The crown of the last premolar and molars are dominated by one labial (protoconid) and two lingual (paraconid and metaconid) cusps linked by crests, forming an acute “V” (Fig. 2), which represent the plesiomorphic trigonid of trechnotherian mammals and primitively the three main lower cusps of postcanine teeth^18,19^. The p2 is slightly mesio-distally larger and labio-lingually narrower than p3; p3 is larger in all dimensions than the molars; m1 is smaller than m2 and m3 and m3 is smaller than m2; thus, m2 is the largest molar. In occlusal view, p2 is roughly triangular with the acute angle facing mesially, p3 is roughly rectangular, with the major axis mesio-distally oriented, m1 and m2 are almost quadrangular, being slightly wider than long, and m3 is trapezoidal, with the longest side facing mesially. In occlusal view, the tooth sequence has a slightly sigmoidal line, with p2 and p3 more labially placed than m2 and m3, the m1 having an intermediate position (Fig. 2). The crown complexity increases posteriorly, with p3 having the same configuration of the molars, and m1 and m2 very similar to each other. The m3 reduces about 1/3 its distal width but keeps the same crown structures than m1–m2 (Fig. 2).

The p2 lacks the portion of the crown mesial to the protoconid. It is a bulbous tooth, with the protoconid occupying most of the crown. It has strong apical wear exhibiting a thick enamel layer and dentine. The labial wall of the paraconid is strongly convex, with a swelling at the crown base. The labial wall is straighter and distolingually invaded by an accessory cingular cusp (Fig. 2). The preprotocristid is stout and slopes down toward the embrasure with the paraconid, which is not preserved. Taking into account the broken base of the crown, a conspicuous paraconid is inferred, as in the p2 of *Coloniatherium*^12,15^. The postprotocristid is sharper and projects disto-lingually to contact the base of the metaconid. This crest also delimits distally a concave surface. There is an extra thin crest over the distolabial surface of the protoconid, which descends to contact the cingular distolabial cusp of the distal cingulum. The metacone is much smaller than the protoconid and apparently smaller than the inferred paraconid (Fig. 2). There is a distal cingulum, separated from the metaconid and protoconid by a deep valley. The distal cingulum runs transversally along two-thirds of its lingual portion and then curves mesio-labially. It has minute crenulations, with a distinctive cusp at its labial end.

The p3 also lacks part of the mesial portion of the crown, but its outline is evident (Fig. 2). The protoconid is moderately procumbent, taller than in any other cheek tooth, and occupies most of the labial half of the crown. The labial surface of the protoconid is less convex than in p2. The pre- and postprotocristid forms an angle of 50º, with the preprotocristid extending mesio-lingually to contact the base of the broken paraconid and the transversely projected postprotocristid. The preprotocristid is slightly inclined and thick at its contact with the paraconid (Fig. 2). Both pre- and postprotocristids enclose a concave basin, which is reduced and partially divided by a fold of the lingual wall of the protoconid. The metaconid is placed close and lingually in front of the protoconid. In lingual view, it is the main dominant cusp, as tall as the protoconid, and considerably taller and larger than the paraconid. The metaconid and protoconid are connected by the postprotocristid, which extends lingually to contact the short distolingual crest of the metaconid; they form an almost right angle. The metaconid has its tip worn out, defining a drop-shaped outline. The swelling of the lingual wall of the crown enlarges considerably the transversal width of the tooth and positions the apex of the metaconid more labially (medially displaced). The p3 has conspicuous mesial and distal cingula, which are mesio-distally broader at their labial side and separated from the wall of the protoconid base by a narrow notch (Fig. 2). The occlusal surface of these cingula is partially eroded, especially the mesial one which is also incompletely preserved. The crown configuration of p3 is similar to that of the molars, mainly differing in that the trigonid has a smaller triangular area than the molars, but the labial and lingual swelling of the crown base plus the mesial/distal cingula result in a larger and bulbous tooth, the largest of the cheek-tooth series. The differences between p2 and p3 are conspicuous, especially in the occlusal outline, less developed cingula, and relative size (protoconid much larger in p2 than in p3) and placement (metaconid is distolingual to protoconid in p2 instead of lingual as in p3) of the trigonid cusps (Fig. 2).

The m1–m3 have a similar morphology, with slight differences in size among them; the m3 is characteristic in having a narrower distal portion than the mesial one. In addition, the swelling of the crown base is stronger in the labial side of m2–m3 than in m1 (Fig. 2). The three molars have a tall protoconid, which is slightly mesio-distally compressed at its labial sector. The pre- and postprotocristids form an angle of ~ 45º in all molars, which is smaller than the angle of p3. The preprotocristids run obliquely and end in the mesiolabial wall of the paraconid; they are straight in m1-m2 and slightly bent in m3. The postprotocristids are transversal to the main dentary axis, being straight in m1–m2 and shorter and bent in m3. The protoconids bear a lingual fold, apically sharp (as seen in m3), which becomes rounded after apical wear (as in m1–m2). The basin defined by the cristids of the protoconid is larger in m2 than in m1 and m3, and its size is considerably affected by wear. The paraconid of each molar is placed at the mesiolingual corner of the crown. It is a conical cusp, considerably smaller and shorter than the metaconid. The separation of paraconid and metaconid is defined by a deep and narrow notch, clearly exposed in the lingual side of m3, less affected by wear. In addition, the paraconid apex is placed higher in relation to the mesial cingulum, defining a tall mesial wall. Along the molars, the paraconid is comparable in size whereas the metaconid decreases backwards. The metaconid is a large cusp, subconical, subtly smaller than the protoconid. Its labial wall is concave whereas the lingual one is almost flat, defining most of the lingual profile of the crown. The metaconid is considerably larger in m1-m2 than in p3, but due to wear, the distal crest of the metaconid that contacts the postprotocristid is not evident in m1-m2. In m3, the distal wall of the metaconid seems to reach the postprotocristid directly. In m1 and m3, there is no evidence of a labial crest on the metaconid, which seems to be absent in m1 due to wear. In m2, the mesiolabial corner of the metaconid bears a worn crest that descends labially to the trigonid basin, but lacks contact with the protoconid fold. The crowns of the molars are flanked by distinctive mesial and distal cingula, extended from the lingual to the labial side (Fig. 2). Both cingula are separated from the trigonid by a continuous groove, which is narrower in m2 than in m1 and m3. The mesial and distal cingula become mesio-distally broad in the labial sector, and especially in m1-m2 they curve towards the protoconid base, thus having a lingual component. The mesial cingulum is better developed than the distal one, especially in m3 in which the distal portion of the crown is transversely narrow. There is intense wear in the cingula of m1–m2, obscuring evidence of crenulations or discrete cuspules. However, the mesial cingulum of m3 has a conspicuous cusp at its labial end, as well as subtle constrictions that suggest tiny cuspules. The mesial cingulum is placed slightly higher than the distal one. Extra cingula or cingular cusps on the labial/lingual surfaces of m1-m3 are absent, contrary to the condition of *Peligrotherium* and *Reigitherium*^16^. Posterior to m3 there is a retromolar space as long as half a molar size (Fig. 2).

The roots of p2-m3 were virtually reconstructed by micro-CT images (Fig. 3). All teeth have two main roots, one in the mesial and the other in the distal half of the tooth. Only p3 has a tiny accessory root. The roots of p2 are unequal, the mesial one being cylindrical and the distal one transversely compressed, tapering to the root apex (Fig. 3). The mesial root is slightly postero-ventrally inclined, and longer and thicker than the distal one. The apex of the mesial root is closer to the ventral edge of the dentary than in any other teeth. The mesial and distal roots of p3 are subequal and both are transversely compressed (Fig. 3). The tiny accessory root is placed anterior to the labial side of the distal root. It is conical, slightly inclined and part of its base collapses with the distal root. The roots of m1–m2 are similar, both transversely compressed, tapering to the apex root. They are parallel to each other and the mesial wall of the distal roots has a longitudinal, shallow groove. The roots of m3 are distinctive in size and shape, concomitant with the crown shape. The mesial root is transversely compressed, tapering to the apex root, and slightly smaller and shorter than in the preceding molars. The distal root has a transversely wide base but tapers abruptly and much of the root is oval in cross-section. In the p2 and molars, the roots are taller than the crown, whereas in p3 they have a similar height (Fig. 3).

*Upper premolar.* CPAP-5008 consists of an almost complete right upper P3 with portions of the maxillary bone around the roots, indicating it was functional at the moment of death (Fig. 4). Most of the parastyle and metastyle cusps are broken, only preserving their bases. Parts of the roots are also broken, missing the distal portion of the lingual root and about 2/3 of the labiodistal one. In occlusal view, the sides of the crown define a roughly rectangular outline, about twice wider than long, with the labial profile slightly convex and the lingual one slightly convex. The crown has intense wear over the paracone apex and its pre- and postparacristae, and the mesial and distal cingula. The paracone is the main cusp occupying most of the lingual half of the crown (Fig. 4). It has a conical shape, slightly mesio-distally flat, and bears the pre- and postparacristae, which defines an acute angle (~ 50º). The preparacrista extends labially, passing the level of the tip of the stylocone, and is separated from the parastyle by a deep and narrow notch. At this point, the preparacrista is slightly thick and exhibits more wear than the postparacrista. The postparacrista extends distolabially to the metastyle, but the crista ends lingually to it, both separated by a wide sulcus. The paracone also bears a short labial crest that contacts the styloconar crest, dividing two small basins (Fig. 4). The stylocone is also a conspicuous conical cusp. Due to wear, it has almost the same height as the paracone. The stylocone is positioned labially and slightly distal to the paracone, at the mesiodistal mid-way of the crown and disconnected of the preparacrista, as occur in most other meridiolestidans (e.g., *Leonardus*, *Reigitherium*, *Mesungulatum*)^11–13,15,23^. On the contrary, dryolestids and paurodontids have a clear connection between the stylocone and the preparacrista^42,45,47^. Both the paracone and stylocone are close to one each other, as in molarized premolars, whereas they become more distant in successive teeth^15^. The labial surface of the stylocone forms the labial surface of the crown, producing a swelling at its base (Fig. 4). Thus, the labial wall of the stylocone has a shallow concave outline in mesial/distal views, instead of being straight or slightly convex as in molars. The styloconal crest is very small and fuses to the labial crest of the paracone at their embrasure. The parastyle is fully disconnected from the stylocone and the preparacrista (Fig. 4). This cusp is not preserved, but its base is somewhat compressed and bears a short cingulum on its labial surface. This small cingulum does not extend over the labial surface and is different from the crenulated and complete labial cingulum of the M1 of *Mesungulatum* and *Coloniatherium*^8,12^. In the P3 of *Coloniatherium*, a labial cingulum is also absent^15^. The broken base of the metastyle is placed labiodistally and close to the stylocone; both are linked by a sharp and short crest developed on its distal wall. Based on its broken base, the metastyle is rounded and placed in a more apical position than the parastyle. The metastyle is separated from the distal cingulum and postparacrista by a deep sulcus (Fig. 4). In contrast, the metastyle and the labial end of the postparacrista are almost in contact in the P3 of *Coloniatherium*^15^. The mesial and distal faces of the crown of CPAP-5008 are flanked by a conspicuous cingulum (Fig. 4). The mesial cingulum extends lingually from above the level of the parastyle to pass the level of the paracone apex, marking off an internal groove. The distal cingulum starts from a flattened labiodistal, small cingular cusp, located distal to the metastylar base, and extends lingually passing the paracone apex. The distal cingulum is slightly mesio-distally narrower and more oblique than the mesial one. Besides, the mesial cingulum seems to be placed closer to the crown base than the distal one. Both cingula have small and worn out crenulations, especially on the lingual side. The continuous mesial and distal cingula in the last premolars and all molars of mesungulatids, such as *Mesungulatum*, *Coloniatherium*, *Peligrotherium*, and *Paraungulathum*, are unique traits of this group^8,11,12,15,24^.

CPAP-5008 has three roots, one lingual and two labial. The lingual root has a subcircular cross-section and lacks most of its distal wall (Fig. 5). It bends slightly labially. The mesiolabial root is oval in cross section, with the main axis transversely oriented. The distal labial root has the largest base, but most of the root is broken. It is sub-rectangular in cross-section, mesio-distally compressed, and about three times wider than long (Fig. 5). Extra-roots were not identified in the micro-CT images.

*Dentary.* The dentary of specimen CPAP-5007 is preserved in two parts, the one holding p2 and a second bearing p3 to m3, with its posterior portion broken backward at the base of the coronoid process. The horizontal ramus of the dentary is robust and transversely broad, to hold the complex system of roots. In labial view, the ventral edge of the dentary is concave, whereas the alveolar one is irregular in its labial side and almost straight in the lingual side (Fig. 2). The lateral wall of the dentary is dorso-ventrally concave, tallest at the level of m3. The lowest preserved point is between p2-p3 but at this part the alveolar edge is only partially preserved. One mental foramen is preserved, here interpreted as the posteriormost one, considering that other foramina pierce the missing anterior portion of dentary. The mental foramen seems to be large, but its posterior edge is broken. It is placed below the crown of p2, near the ventral edge (Fig. 2). A tiny nutrient foramen is also observable at the base of the coronoid process, near the anterior edge of the masseteric fossa. The labial alveolar border exhibits an irregular line, with the inter-radicular processes less developed than the inter-alveolar ones. Only the anterodorsal portion of the masseteric fossa is preserved, being deeper just posterior to the base of the coronoid process. The coronoid process is almost completely lost, preserving its base, which is transversely wide and antero-posteriorly short, forming an antero-laterally rounded edge. The anterior surface of the coronoid base is slightly concave and faces anteriorly, suggesting the anterior edge of the coronoid process was vertical. The base of the coronoid process is also placed in front of the last molar, separated by a conspicuous retromolar space. The medial wall of the dentary is almost flat and considerably taller than the lateral side. There is a shallow longitudinal depression placed at mid-height below the cheek teeth (Fig. 2), which may indicate the remnant surface of Meckel’s cartilage that is lost during ontogeny^55,56^. Scars for a coronoid bone or other postdentary bones are not evident and details of the posterior portion of the dentary (e.g., angular process, condyle) are not preserved.

#### Replacement sequence

**Figure 6:**
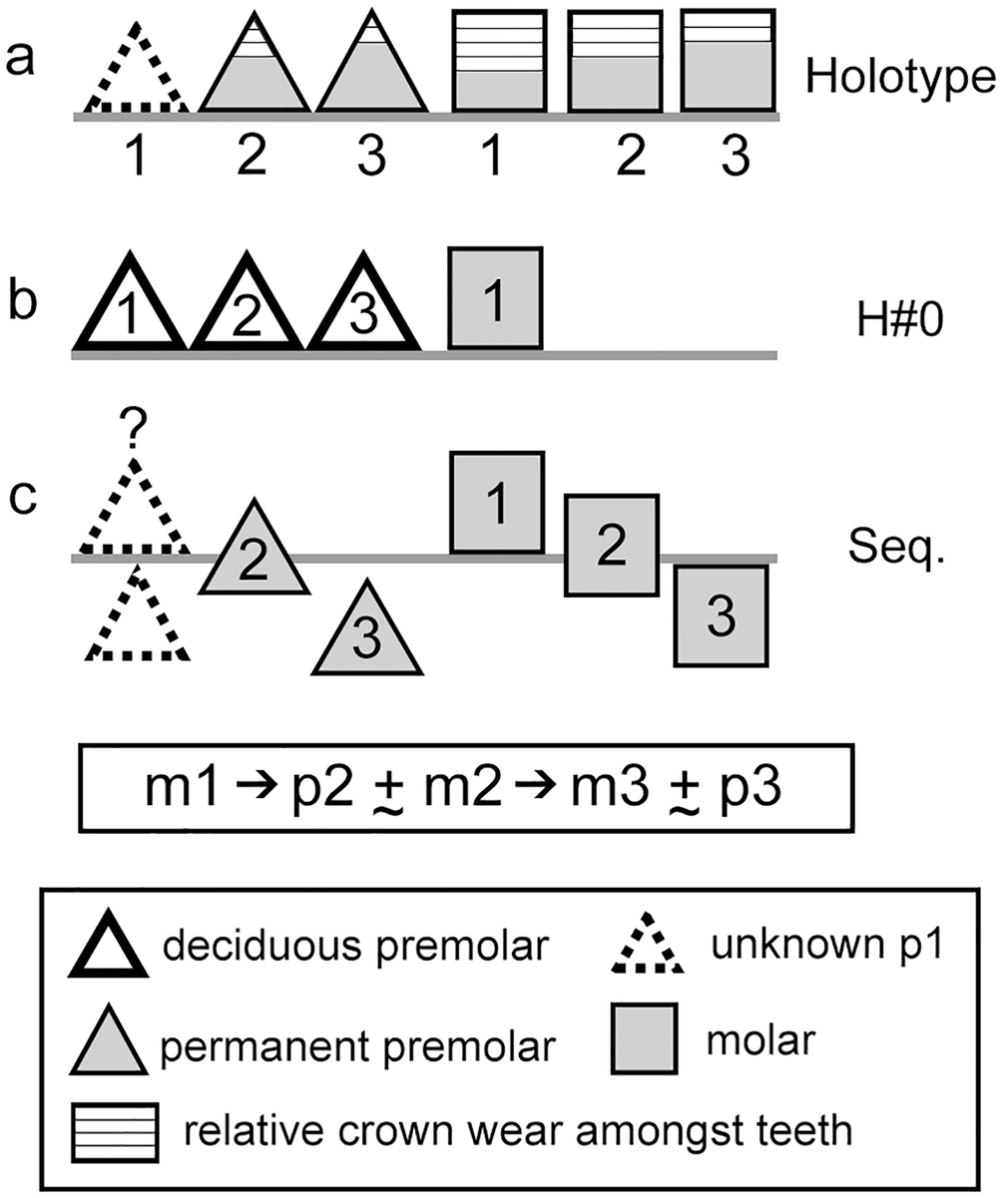
Lower cheek teeth replacement sequence in *Orretherium tzen* gen. et sp. nov. (**a**) Relative crown wear on preserved cheek teeth of CPAP-5007. (**b**) Hypothetical stage of a juvenile, with deciduous premolars and first molar (H#0). (**c**) Inferred sequence of replacement based on (**c**).

Inferences on the tooth eruption sequence of *Orretherium* can be drawn with the available material using the relative wear over the crown as a proxy (Fig. 6). In addition, we assume a diphyodont replacement of premolars for *Orretherium*, a typical condition among trechnotherians^57–59^, and the definition of molar as any tooth posterior to the last postcanine showing evidence of replacement, as amended by Bi et al.^60^, based on Owen^61^. The crown wear in CPAP-5007 is as follows: (i) m1 has the most worn crown; (ii) p2 and m2 have both a roughly similar tooth wear stage, stronger than p3 and m3; (iii) the wear of m3 is roughly similar or slightly stronger than that of p3; and (iv) the cervix of p3 is in a lower position than the remaining teeth (not fully erupted) (Fig. 6). Based on this, the following sequence is inferred: (i) m1 erupted before than p2-p3, being functional with the deciduous (d) premolars; (ii) dp2 was replaced earlier than dp3, likely simultaneous with eruption of m2; (iii) dp3 was replaced at the same time or slightly later than eruption of m3; (iv) possibly p3 was the last tooth to fully erupt, slightly later than m3; and (v) wear facets are conspicuous before the crowns were fully erupted, as seen in the incomplete erupted p3 (Fig. 6). This model indicates an anterior to posterior sequence of replacement for p2-p3 (due to the lack of replacement evidence for p1, we cannot extend this conclusion to the entire premolar series), and an anterior to posterior eruption of molars. Based on this model, the p2 and the molarized p3 are considered permanent premolars, and the three posterior teeth molars, as inferred for *Peligrotherium*^24^ and *Coloniatherium*^12^. Although we cannot refer the P3 of CPAP-5008 unambiguously as part of CPAP-5007, the crown wear of the P3 is similar to the p3, supporting that CPAP-5008 is a permanent molarized premolar. Stronger wear on P3 could certainly indicate a different individual, but this is not the case.

### Phylogenetic relationships

**Figure 7:**
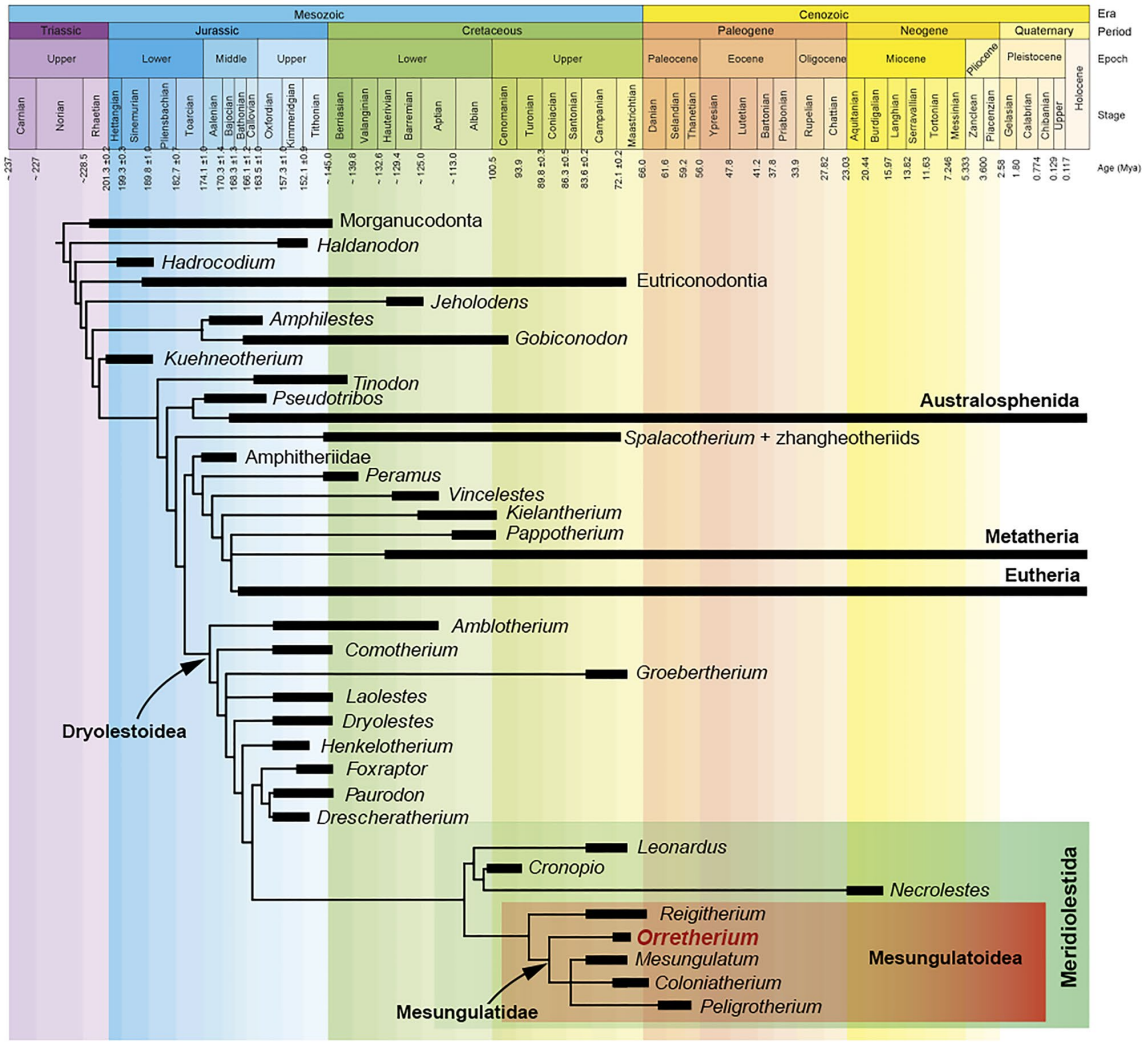
Time-calibrated phylogenetic trees. Simplified strict consensus tree of 12 MPTs with the position of *Orretherium tzen* among meridiolestidan mammals.

Our parsimony phylogenetic analysis resulted in 12 most parsimonious trees of 1196 steps (Ci = 0.372, Ri = 0.728). A simplified strict consensus tree is shown in Fig. 7 (see also Supplementary Data 1). *Orretherium* is nested within Meridiolestida, as sister-taxon of an unresolved clade comprising *Mesungulatum*, *Coloniatherium*, and *Peligrotherium*; these four genera are considered the clade Mesungulatidae (Fig. 7). *Mesungulatum* is the least known taxon of the group, lacking data on most of the premolar morphology as well as the jaw and the rest of skeleton. Except for MACN-RN 06, a left dentary with m1 and m2, tooth sequences are interpreted based on isolated teeth^15,16^. *Reigitherium* is placed as sister taxon of mesungulatids, as the basalmost Mesungulatoidea. Most of the meridiolestidan clades have high Bremer support values (see Fig. S6). The crown and root dental gross morphology of the P3 and p2-m3 of *Orretherium* follows the typical pattern of mesungulatids, but some traits, such as the lack of supernumerary roots in P3 and p2 and the relatively small size of p2, place the new Chilean taxon as basal to the Late Cretaceous *Mesungulatum* and *Coloniatherium* and the early Paleocene *Peligrotherium*.

**Figure 8:**
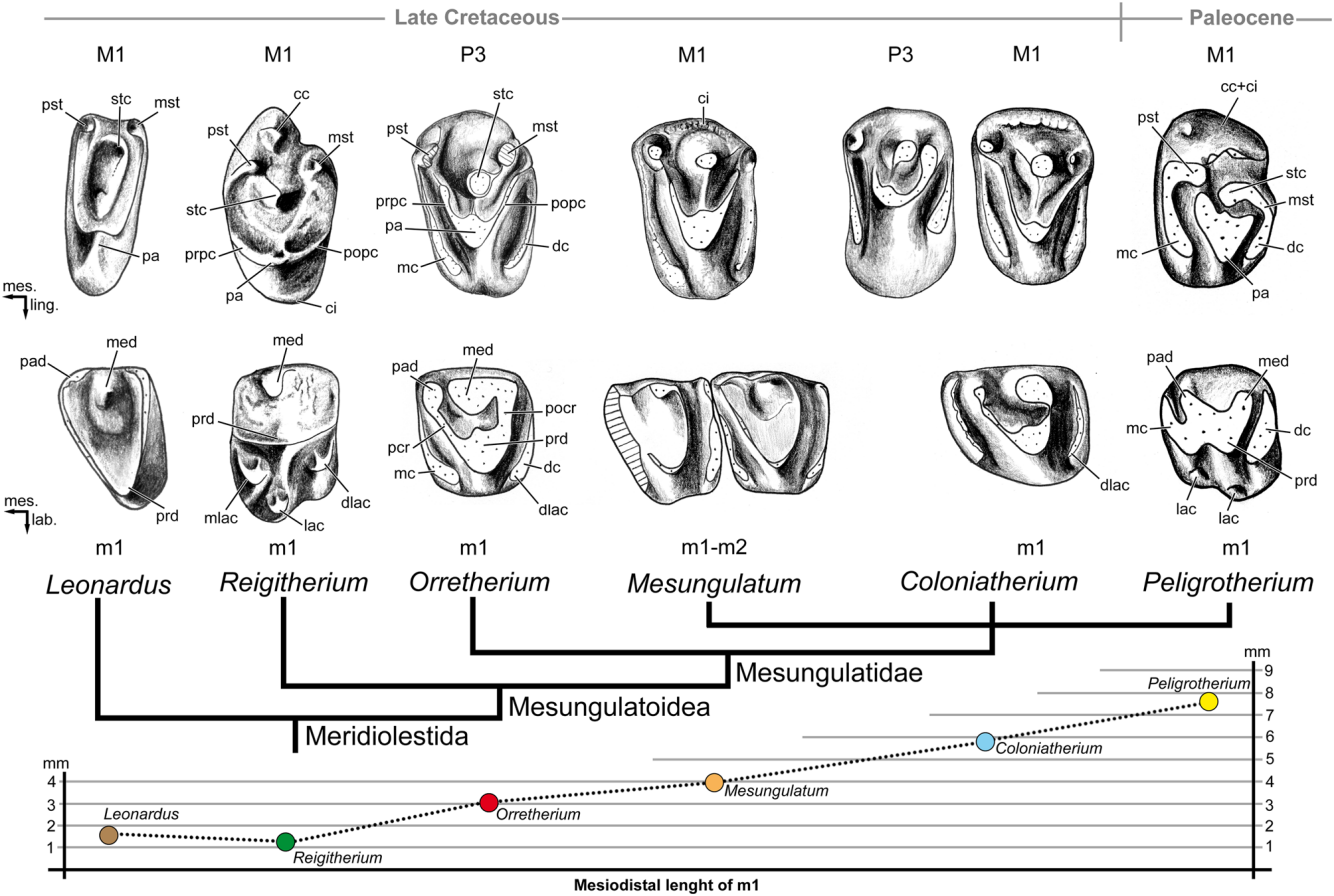
Comparison of selected left upper/lower postcanine teeth of some meridiolestian mammals and their size based on m1. *Leonardus* is based on MACN-RN 172 (M1) and MACN-RN 1097 (m1), as interpreted by Rougier et al.^15^; *Reigitherium*, Harper et al.^16^: Fig. 3a and MPEF-PV 2238, M1; MPEF-PV 2317, m1); *Orretherium*, CPAP-5008 (P3) and CPAP-5007 (m1); *Mesungulatum*, MACN-RN 03 (M1) and MACN-RN 06 (m1-m2); *Coloniatherium*, MPEF-PV 2081 (P3), MPEF-PV 2078 (M1) and MPEF-PV 2064 (m1), as interpreted by Rougier et al.^12,15^; and *Peligrotherium*, MPEF-PV 2351, as interpreted by Páez Arango^24^ and Rougier et al.^15^. See also Fig. S3. For comparative purposes the M1/m1 of *Leonardus*, m1 of *Reigitherium*, P3 of *Reigitherium*, and P3 of *Coloniatherium* are inverted. Drawings made by A.G.M. *cc* cingular cusp, *ci* cingulum, *dc* distal cingulum, *dlac* distolabial cingular cusp, *mc* mesial cingulum, *med* metaconid, *mlac* mesiolabial cingular cusp; *lac* labial cingular cusp, *mst* metastyle, *pa* paracone, *pad* paraconid, *pcr* preprotocristid, *pst* parastyle, *pocr* postprotocristid, *popc* postparacrista, *prd* protoconid, *prpc* preparacrista, *stc* stylocone.

Optimization of some character-states is also difficult to address, considering that the distal upper/lower premolars of *Mesungulatum* and the upper molars of *Orretherium* are unknown. *Reigitherium*, originally related to dryolestoids in its own family Reigitheriidae^9^ and then to docodontans^62^ (an early mammaliaform group), has gained support as a meridiolestidan, particularly as a mesungulatoid, since the discoveries and reinterpretation of several specimens from La Colonia Formation^12,13,16^. However, Harper et al.^16^, based on a reduced dataset of taxa (#10) and characters (#44), obtained a clade with *Reigitherium* plus *Peligrotherium* as sister-group of *Mesungulatum* plus *Coloniatherium*. Our results do not support the inclusion of *Reigitherium* within mesungulatids, or a clade with *Peligrotherium* (see the Discussion) and supporting instead its position as sister taxon of mesungulatids, as in previous studies^13,14,31,63^. This results from changes in some character-state coding for *Reigitherium* that differ from the analysis conducted by Harper et al.^16^. The “high grade” of bunodonty in *Reigitherium* and *Peligrotherium* was the main aspect that drives their sister-taxon position in Harper et al.^16^. However, comparisons of the tooth characters of both taxa shows remarkable differences (Fig. 8), and mesungulatids (including *Peligrotherium* and excluding *Reigitherium*) clearly highlight a morphological trend in the dentition, with similar tooth crown topologies that increase in size and bunodonty (see below). The presence of four premolars in *Reigitherium* (as expressed by Harper et al.^16^), together with its small size and its distinctive crown configuration may likely indicate another peculiar radiation of South American mammals, still poorly known but certainly close to mesungulatids.

Another difference obtained regarding previous hypotheses^13,49^ is the paraphyletic condition of dryolestids at the base of the Dryolestoidea clade (Fig. 7). Furthermore, we obtained a “paurodontid” clade (*Foxraptor*, *Paurodon* and *Drescheratherium*) as sister-group of Meridiolestida as in some previous analyses^13,14^, instead of being early diverging dryolestidans^49^. Our result supports a Dryolestoidea clade as proposed in Rougier et al.^13^, that was not obtained in other studies^14,31^. Certainly, changes in character-scorings, the inclusion of the new taxon, and the incompleteness of several taxa may explain this result, which may be reversed by new fossils and larger datasets. Furthermore, the morphological and temporal gaps between meridiolestidans and putative dryolestidans from the mid-to-Late Cretaceous of Gondwana and the Jurassic-Early Cretaceous of Laurasia indicate a still undeciphered history. The phylogenetic proposal by Averianov et al.^49^, in which meridiolestidans are placed as non-cladotherian trechnotherians, is not supported by our analysis, but larger datasets coupled with more complete specimens and more plesiomorphic South American meridiolestidans may support different evolutionary scenarios in the future.

## Discussion

*Orretherium tzen* gen. et sp. nov. from the Late Cretaceous of southern Chile represents the second mammal species for the Dorotea Formation (Magallanes Basin) and the southernmost record of a Mesozoic dryolestoid. Within the abundance of meridiolestidan mammals in terrestrial faunal associations of central and northern Patagonia that predate the end of the Mesozoic, the occurrence of *Orretherium* in southern Patagonia expands the distribution of this group further south, being at a palaeolatitude of ~ 54º S during the Late Cretaceous^64^. During the Late Cretaceous, since the Campanian, the Magallanes region was palaeogeographically very close to the West Gondwana margin^65^ and separated from the amalgamate West Antarctica crustal block (Antarctic Peninsula)^66^. However, temporal land bridges through the Scotia Arc could facilitate intercontinental dispersion of organisms^67^. Therefore, the Magallanes region and the Antarctic Peninsula could have been part of a unique domain, bearing a typically Weddellian (Southwestern Gondwana) biota, including gondwanatherians and meridiolestidans. The *Orretherium* bearing horizon of the Dorotea Formation (Magallanes Basin) seems to be coeval with horizons of the Gamma (= Herbert Sound) Member of the Snow Hill Island Formation (late Campanian-early Maastrichtian, James Ross Basin, Larsen Basin) of the Antarctic Peninsula crustal block. This palaeogeographic proximity and the record of an Eocene non-therian cladotherian in the Antarctic Peninsula^29^ suggest a still hidden southern history for this group by the Late Cretaceous to Paleogene.

**Figure 9:**
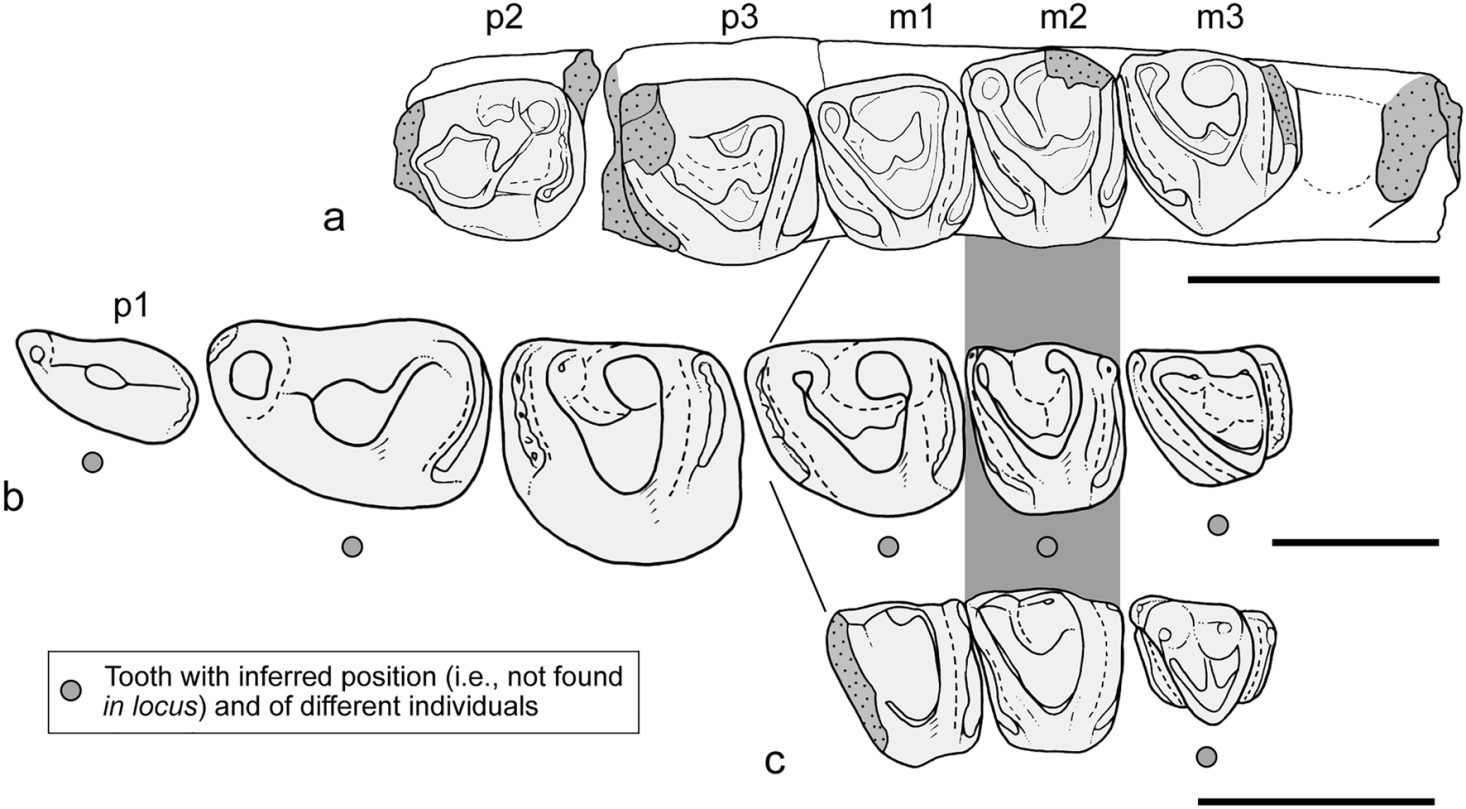
Comparison of lower postcanine teeth among selected mesungulatid mammals. (**a**) *Orretherium tzen* gen. et sp. nov. (CPAP-5007, holotype). (**b**) *Coloniatherium cilinskii*, premolar-molar sequence reconstructed upon different individuals. (**c**) *Mesungulatum houssayi*, molar sequence based on two specimens. Drawings made by A.G.M.; (**b**,**c**) based on Harper et al.^16^ and Rougier et al.^15^. They are scaled by the mesiodistal length of m2. Scale bar: 5 mm.

*Orretherium* is noticeably similar to three species recovered in the Los Alamitos (*Mesungulatum houssayi*^8^), Allen (*M. lamarquensis*^11^), and La Colonia (*Coloniatherium cilinskii*^12^) formations and to some extent to the early Paleocene *Peligrotherium*, which achieved larger body size and more bunodont cheek teeth^16,24,27^. The main features shared by these taxa include: a well-developed labio-lingually extended mesial and distal cingula on upper and lower molariforms, with occlusal contact between opposite teeth; the presence of mesio-distally compressed roots in last premolars and molars; the presence of small extra-roots in the last premolars (at least one extra root in the p3 of *Orretherium* and unknown in *Mesungulatum*); enlargement of the last premolar (unknown in *Mesungulatum*); and presence of three premolars (unknown in *Mesungulatum*, inferred in *Orretherium*) and three molars (Figs. 8, 9).

The inferred lower postcanine series in *Coloniatherium* and *Mesungulatum* were taken from Harper et al.^16^ and Rougier et al.^15^ (see Fig. 9), which are based on a pool of specimens collected in their respective type localities. Certainly, they illustrate a reliable postcanine series, also supported by isolated, mostly edentulous jaws of *Coloniatherium*^12^ and the well-preserved and more complete specimens of *Peligrotherium*^15,16,24,27^. Data on *Mesungulatum* is much more limited than for *Coloniatherium* and *Orretherium*, especially regarding the premolar positions. Some considerations can be made on those reconstructed series taking into consideration the *in locis* postcanines of *Orretherium*. We suggest that the inferred m1 (MEPF-PV 2064) and m3 (MPEF-PV 2138) of *Coloniatherium* may be of a different, closely related taxon, considering that m1 and m2 of *Orretherium* and *Mesungulatum* are remarkably similar in morphology (Fig. 9). Specimen MEPF-PV 2064 referred to m1 of *Coloniatherium* could represent the p3 of another smaller mesungulatid species, considering that is relatively longer mesio-distally than the molars of *Mesungulatum* and *Orretherium*. Similarly, the m3 (MACN-RN 06) referred to *Mesungulatum* could represent a last molar of another taxon, perhaps a lower tooth of *Paraungulatum* or a still undescribed species, taken into consideration the large size of the paraconid related to the metaconid and its much smaller size compared to the m1-m2, preserved *in locis*^8^ (Fig. 9).

Comparison of the P3 is not possible with *Mesungulatum* since this position is unknown. The P3 of *Orretherium* is more molarized than the P3 of *Coloniatherium* in having: longer post- and preparacristae; metastyle more labially placed; distal cingulum more lingually expanded; and a less reduced swelling of the lingual base of the paracone. In *Coloniatherium*, the P3 is more bulbous as is also the case of the p3^12,15^. The P3 of *Orretherium* has apico-basally shorter mesial and distal cingula than that of *Peligrotherium* and the main crown cusps are clearly defined in the Chilean taxon, which is not the case of the latter. The micro-CT images did not reveal extra-roots in the P3 of *Orretherium*, differing from the condition of *Coloniatherium* and *Peligrotherium*^12,15,16^.

Data on tooth replacement sequence in mesungulatids is limited mainly because of the lack of juveniles and more complete specimens. However, previous studies^24^ mentioned that the last premolar of *Peligrotherium* has less wear than the penultimate premolar and first molar, indicating it was replaced. A similar condition is observed in the lower jaw of *Orretherium* (Fig. 6), marking the boundary between molars and premolars. The well-documented dental replacement in the lower premolars of *Dryolestes* follows an alternating pattern in a sequence: p1–p3–p2–p4^58^, as is also similar in zhangheotheriids and possibly spalacotheriids (although the number of premolar teeth varies among species), representing the plesiomorphic condition for trechnotherians^59,60^. Mesungulatids have a reduced 3p/3 m count, contrasting with the 4p/8-9 m of dryolestids (e.g., *Dryolestes*, *Amblotherium*, *Laolestes*^41,42,45^), the 2-3p/4-5 m of paurodontids (*Paurodon*, *Drescheratherium*, *Foxraptor*^45,68,69^), the 5p/6-?7 m of spalacotheriids (e.g., *Spalacolestes*, *Akidolestes*, *Lactodens*^70–72^), and the varied count (1-4p/4-6 m) in zhangheotheriids (e.g., 3p/5–6 m, *Zhangheotherium*; p1-3/m6 *Maotherium*; 4p/4 m, *Anebodon*^60,73–75^). Considering four premolars as the plesiomorphic condition for dryolestoids, and assuming that the first premolar was lost in mesungulatids, a similar pattern in the eruption sequence between *Orretherium* and *Dryolestes* cannot be completely discarded: (1º) p1 (*Dryolestes*) = p0 (first plesiomorphic premolar lost in *Orretherium*); (2º) p3 (*Dryolestes*) = p2 (*Orretherium*); 3º); p2 (*Dryolestes*) = p1 (not preserved in *Orretherium*); 4º) p4 (*Dryolestes*) = p3 (*Orretherium*). In *Dryolestes* the last premolar erupted just prior to the m6^58^. Considering the reduced number of molars (three) in mesungulatids, and that the last molar erupted slightly before the last premolar in *Orretherium* (Fig. 6), we interpret a heterochronic delay of molar eruption in mesungulatids with regard to basal dryolestoids. Furthermore, the almost simultaneous eruption of the last premolar with the last molar recalls the condition of therians^76^, differing from dryolestids, paurodontids, spalacotheriids, and zhangheotheriids, which possess a higher number of molars.

Rougier et al.^11^ was the first to use Mesungulatidae (erected by Bonaparte^8^) for a clade encompassing *Mesungulatum*, *Coloniatherium*, *Reigitherium*, and *Peligrotherium*; the two latter genera originally allocated within Reigitheriidae and Peligrotheriidae, respectively. Later, Rougier et al.^13^ defined Mesungulatoidea, as the last common ancestor of *Reigitherium*, *Mesungulatum*, and *Peligrotherium* plus all its descendants. A basal placement for *Reigitherium* among mesungulatoids has been recovered in most phylogenies^13,14,31^. Considering the upper molars, Bonaparte^23^ positioned *Reigitherium* (he used the holotype MACN-RN 173 as an upper molar, which is now considered a lower molar^16,62^) as “derived” from a *Mesungulatum* pattern. Further, phylogenies of Averianov et al.^49^ and Harper et al.^16^ nested *Reigitherium* as sister-taxon of *Peligrotherium*. The few modifications we made in the scoring of some character-states for *Reigitherium* (see Supplementary Data 1), mostly based on the recently published specimens^16^, support previous phylogenetic studies in which it occupies a basal position within mesungulatoids (Fig. 7).

We note discrepancies in the interpretation of some structures of the cheek teeth presented by Harper et al.^16^. The upper molars of *Reigitherium* lack the conspicuous labio-lingually extended mesial and distal cingula present in *Mesungulatum*, *Coloniatherium*, *Paraungulathum*, *Peligrotherium*, and *Orretherium* (based on the molarized P3), which fully flank the primary trigon (Fig. 8). Instead, the bowed and conspicuous pre- and postparacristae define the mesial and distal margin of the crown contacting the mesiolabial parastyle and distolabial metastyle, with the stylocone located in between and slightly lingual to both stylar cusps^16^ (Fig. 8). Only a small lingual cingulum can be observed in the upper molars of *Reigitherium* (Fig. 8). In addition, with the exception of the neomorphic ectostyles in the labial side of M1 and M2 of *Reigitherium*, which are autapomorphic^16^, together with the lack of complete mesial and distal cingula and the bowed pre- and postparacristae, the crown shape is rather similar to some non-mesungulatid meridiolestidans, such as *Leonardus*^9,77^ or *Casamiquelia* (a meridiolestidan of still uncertain affinities^9,10,15^), than to mesungulatids (Fig. 8). Regarding the lower molars, *Reigitherium* differs from mesungulatids in the absence of: (i) mesial and distal cingula fully extended labiolingually, (ii) a paraconid and a noticeable oblique preparacristid, and (iii) a postparacristid transversely linked with the metaconid. Moreover, the lower molar crown pattern of *Reigitherium* has well-developed mesiolabial and distolabial cingular cusps together with other accessory labial cusps^16^, which result in distended labial platform representing about 1/3 of the tooth width (Fig. 8). Although the mesiolabial and distolabial cingular cusps of *Reigitherium* are likely homologous to the labial thickening and cusp (only seen in unworn teeth) of the mesial and distal cingula of mesungulatids, we must be cautious in considering that they have the same character-state as was scored in previous studies^14,16^. In mesungulatids, the mesial and distal cingula form a shelf that extends along most of the mesial and distal sides of the trigonid and are not restricted to the labial side. The structure of the trigonid in *Reigitherium* is also noticeably different to that of mesungulatids, being more rectangular than triangular (i.e., mesungulatids), plus the lack of the aforementioned traits (i-iii). The number of four premolars in *Reigitherium*^16^ *versus* three in mesungulatids^12,13,15,24^ and the relative size of the last premolars compared to the size of molars (mesungulatids have large last premolars) are also traits that differentiate them.

Consequently, these new interpretations resulted in the basal placement of *Reigitherium* among mesungulatoids, contrasting with the last phylogenetic hypothesis presented by Harper et al.^16^. The interpretations of the cheek teeth for *Reigitherium*, as shown in this study, suggest that this taxon may represent a still unrecovered radiation of very small meridiolestidan mammals, which shared a common ancestor with mesungulatids. If so, the tendency towards bunodonty was acquired more than once within South American meridiolestidans. Our result supports the Mesungulatidae clade as constituted by the Late Cretaceous *Orretherium*, *Mesungulatum*, and *Coloniatherium*, and the early Paleocene *Peligrotherium*. These taxa show an increase in size (see Fig. S4), which is greatest in *Peligrotherium*, with also an extreme bunodont dentition^15,16,24,27^. The placement of *Paraungulatum* among mesungulatids cannot be ruled out considering the crown shape including extended mesial and distal cingula^10,22^, but further specimens are needed to confirm it.

With the initial discovery of the gondwanatherian *Magallanodon*^7^, and now the mesungulatid *Orretherium*, the Late Cretaceous terrestrial faunas of the Dorotea Formation in southern Chile bolster a supra-generic mammalian homogeneity for Patagonia just before the end of the Mesozoic Era. Jurassic and Early Cretaceous mammals from this portion of South America exhibit a distinctive scenario, formed by australosphenidans, eutriconodonts, amphilestids, and zatherians, which were replaced by the Late Cretaceous dominance of gondwanatherians and meridiolestidans, with few putative dryolestid-like forms and possibly multituberculates^15^. Taphonomic biases and/or lack of systematic fieldwork focused on mammal fossils could be responsible for this tendency in the fossil record, but the heavily sampled associations in central and northern Argentinian Patagonia together with this one from southern Chile support a homogeneous mammalian fauna with numerically abundant gondwanatherians and meridiolestidans, over other archaic groups, and eventually therians. Findings of new fossiliferous sites, not only in Patagonia but also in the Antarctic Peninsula and the rest of South America are needed to assert if Patagonia summarizes the fossil record of the continent, or even of Gondwana, or if it is only a small piece of a marvelous history at the dusk of the Mesozoic Era. Certainly, Patagonia was an evolutionary laboratory in which disparate body sizes and craniodental morphologies appeared and predated the establishment of the Cenozoic faunas dominated by metatherian and eutherian mammals.

## Methods

### Fossil specimens, geologic context, and radiometric age

The studied specimens of *Orretherium tzen* gen. et sp. nov. are housed at the Palaeontological Collection of Antarctica and Patagonia of the Instituto Antártico Chileno, Punta Arenas city, Chile, under the acronym CPAP. Casts of the specimens were also deposited at the Red Paleontológica U-Chile of the Laboratorio de Ontogenia y Filogenia, Departamento de Biología, Facultad de Ciencias, Universidad de Chile, Santiago, Chile, and at the Museo Nacional de Historia Natural, Santiago, Chile. Measurements of the studied specimens are detailed in Supplementary Data 1.

The specimens were collected during picking at the Mammal Quarry as well as picking of concentrate generated after screen washing of the sediments. The holotype CPAP-5007 was fragmented in several pieces, which were found during both collecting processes, within an area of around 100 m^2^. The holotype and hypodigm specimens of *Magallanodon baikashkenke* were also collected at the same quarry^7^.

The Mammal Quarry comprises a fossiliferous horizon located around 30 m above the base of Dorotea Formation exposed in the eastern flank of Río de Las Chinas Valley, Cerro Guido Farm, in Magallanes Region. Sediments of Dorotea Formation (upper Campanian–Danian) fill the Magallanes (= Austral) Basin which was a retroarc basin active during the Late Cretaceous-Neogene lapse^78,79^, and represents a transitional shallow marine shelf-edge to tide-dominated delta environment^80–82^. The fossil-bearing mammal horizon comprises sandy mudstones with fine-grained sandstone lenses, representing a floodplain facies associated to a meandering fluvial deposit^80^. The age of this level can be constrained to late Campanian-early Maastrichtian on the base of detrital U–Pb zircon data, which provides values between 71.7 ± 1.2 Ma and 74.9 ± 2.1Ma^83^.

Comparisons with other mammals were based on direct observation of specimens housed at MACN-Pv (RN, Colección Río Negro; Sección Paleontología Vertebrados, Museo Argentino de Ciencias Naturales “Bernardino Rivadavia”, Buenos Aires, Argentina) and MPEF-PV (Paleontología de Vertebrados, Museo Paleontológico Egidio Feruglio, Chubut, Argentina), as well as bibliographical sources cited along the text.

### Micro-CT scanning and processing

The specimens CPAP 5907 and CPAP 5908 were scanned at the SkyScan 1278 scanner owned by the Plataforma Experimental Bio-CT of the Universidad de Chile (Santiago, Chile) using a source voltage of 65 kV and a current of 718 µA, with a voxel size of 50 µm, generating TIFF files in both cases. They were scanned together and posteriorly separated in three data packages (CPAP 5907 is in two parts). The images were segmented with the software 3D Slicer and three-dimensional models were generated with the same software.

### Nomenclatural acts

This published work and the nomenclatural acts it contains have been registered in ZooBank, the proposed online registration system for the International Code of Zoological Nomenclature. The LSID for this publication is urn:lsid:zoobank.org:pub:63B626DC-E3E5-44C6-85E5-D796AB02343A, and the LSIDs for the new erected taxa are: urn:lsid:zoobank.org:act:0C2AF2FA-AAB8-4CBE-AAFC-4082818E8C22 (*Orretherium*), and urn:lsid:zoobank.org:act:FFD7DFEF-4BD3-4109-A5C6-EB9AE70506AC (*Orretherium tzen*).

### Parsimony phylogenetic analysis

To test the phylogenetic affinities of *Orretherium tzen* gen. et sp. nov. we added its holotype and hypodigm specimens to the phylogenetic data matrix of Rougier et al.^14^ plus four characters and coding changes provided by Harper et al.^16^. The modifications of scorings for *Necrolestes* by Wible and Rougier^31^ were also included. Additionally, character-states were modified for some meridiolestidans based on our observations (see Supplementary Data 1). The final data matrix results in 59 terminal taxa and 321 characters of dental, cranial, and postcranial information. The data matrix (Supplementary Data 2) was analyzed under equally weighted maximum parsimony using TNT v.1.5 (Tree analysis using New Technology)^84^. For the analysis 48 characters were considered as additive (ordered): 2, 5, 27, 40, 42, 49, 55, 56, 57, 65, 78, 82, 83, 93, 100, 101, 114, 115, 120, 126, 134, 144, 146, 155, 171, 178, 184, 186, 187, 201, 207, 209, 228, 230, 231, 237, 240, 242, 244, 273, 276, 277, 281, 287, 289, 291, 294, and 299 (sensu Rougier et al.^13^). The search strategies started using traditional heuristic search of 1000 replicates of Wagner tree followed by TBR branch swapping. The best trees obtained were subjected to a final round of TBR branch swapping to find all MPTs. Decay indices (Bremer support values) for nodes are provided in Supplementary Data 1.

## Supplementary Information

Supplementary Information 1.

Supplementary Information 2.

## Data Availability

Additional information, including the dataset analysed in this study is available in the Supplementary Information files.
