# Supplementary material for: New cladotherian mammal from southern Chile and the evolution of mesungulatid meridiolestidans at the dusk of the Mesozoic era
Source: Sci Rep. 2021 Apr 7;11:7594. doi: 10.1038/s41598-021-87245-4 (PMC8027844; doi:10.1038/s41598-021-87245-4)
Supplement: Supplementary file 1 — Supplementary Information 1. [file 41598_2021_87245_MOESM1_ESM.docx]

**Supplementary Data for:**

New cladotherian mammal from southern Chile and the evolution of mesungulatid meridiolestidans at the dusk of the Mesozoic Era

Agustín G. Martinelli*, Sergio Soto-Acuña*, Francisco J. Goin, Jonatan Kaluza, J. Enrique Bostelmann, Pedro H. Fonseca, Marcelo A. Reguero, Marcelo Leppe & Alexander O. Vargas

*Correspondence and requests for materials should be addressed to A.G.M. ([agustin_martinelli@yahoo.com.ar](mailto:agustin_martinelli@yahoo.com.ar)) and S.S.-A. ([sesotacu@ug.uchile.cl](mailto:sesotacu@ug.uchile.cl))

**List of content:**

1. Additional figures of *Orretherium tzen* gen. et sp. nov.
2. Tooth measurements of specimens of *Orretherium tzen* gen. et sp. nov.
3. Tooth measurements of m1 of *Orretherium tzen* gen. et sp. nov. compared to other meridiolestians.
4. List of modifications to the data matrix of Rougier et al. (2012).
5. Character list used in the phylogenetic analysis.
6. Additional data of phylogenetic analysis results.
7. Bibliography for Supplementary Data

**1. Additional figures** ***Orretherium tzen* gen. et sp. nov.**


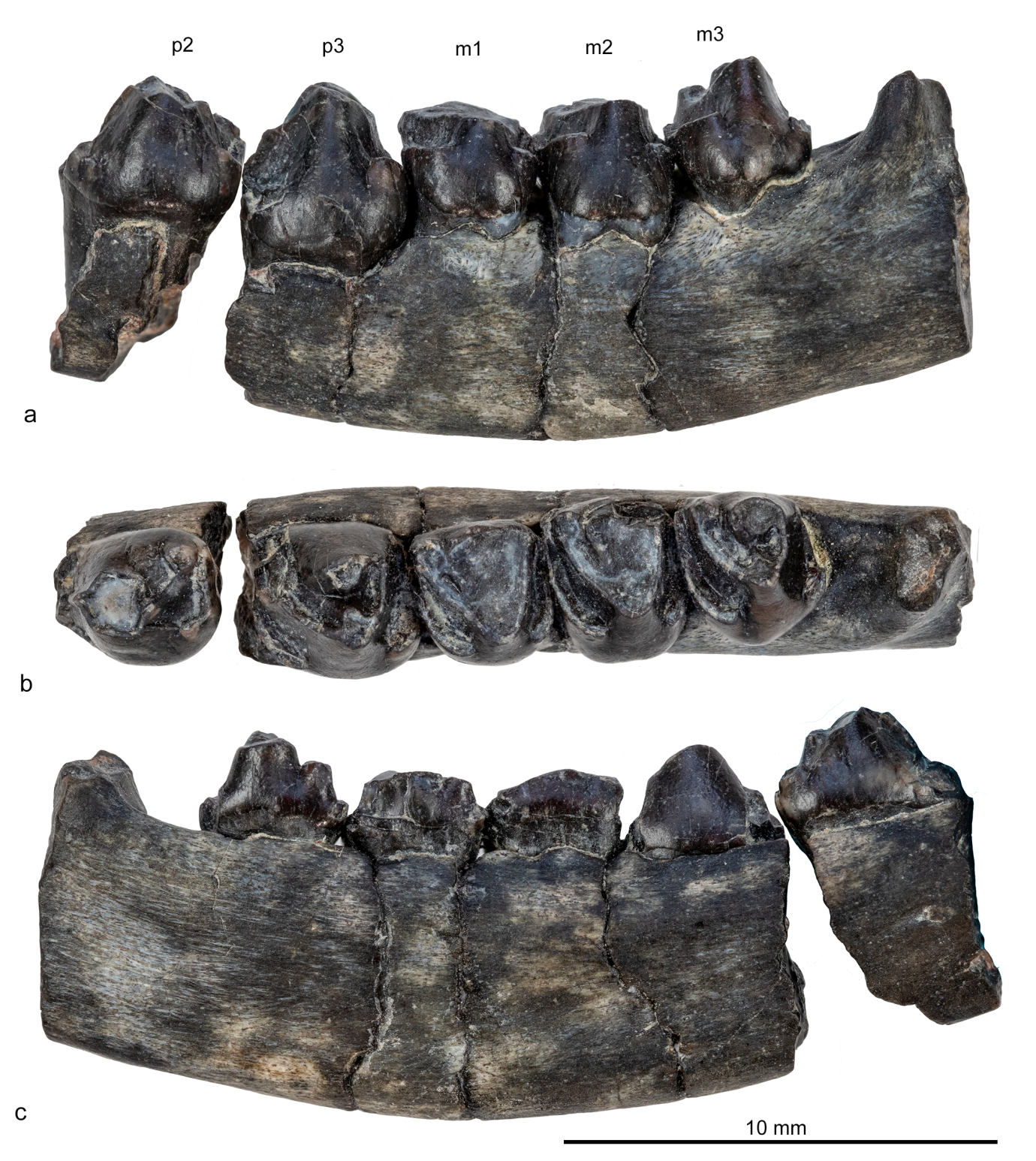


**Figure S1.** *Orretherium tzen* gen. et sp. nov. (CPAP-5007, holotype). a-c, Partial left dentary with p2-p3 and m1-m3 in lateral (a), occlusal (b) and medial (c) views.

**
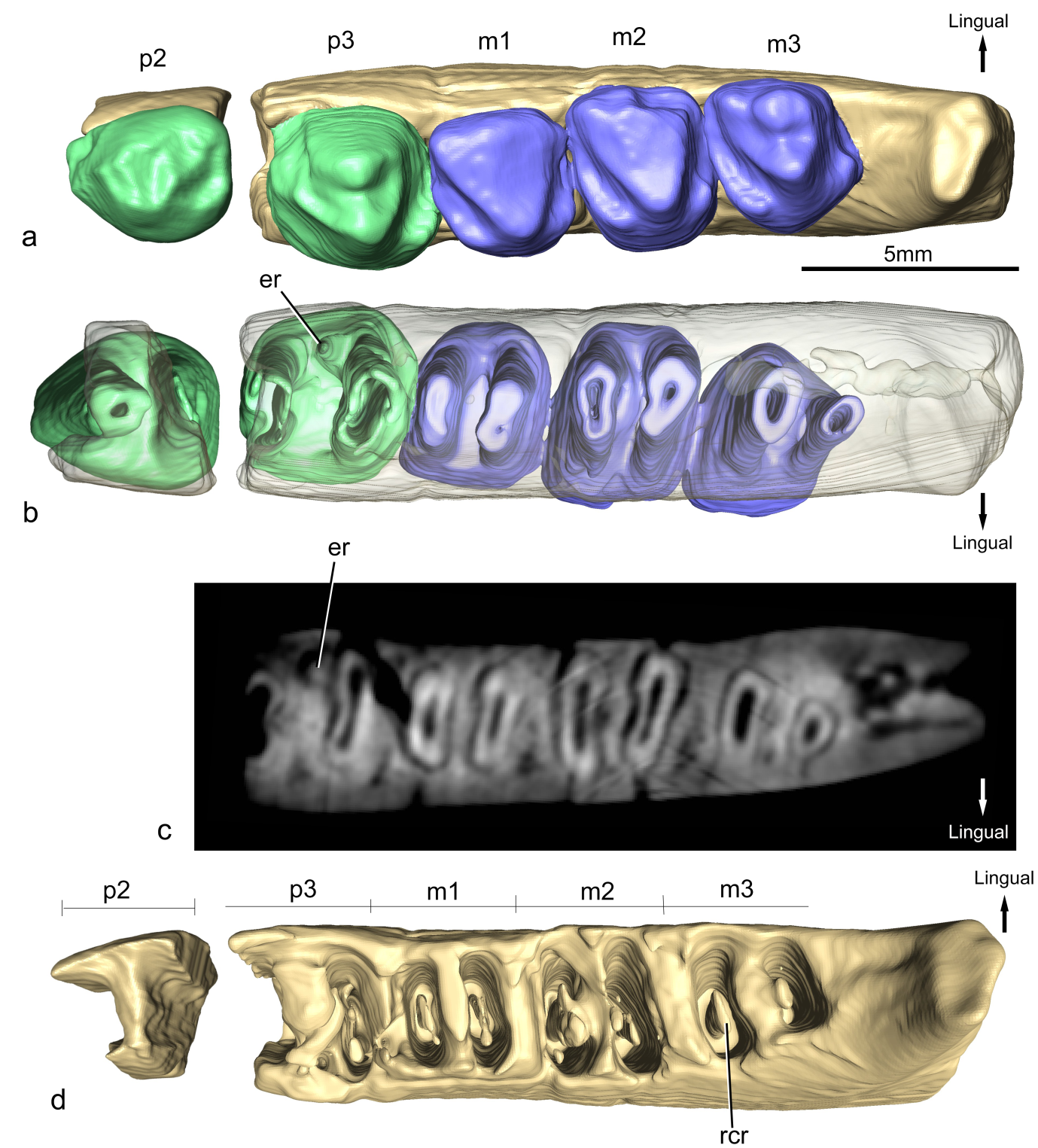
**

**Figure S2.** *Orretherium tzen* gen. et sp. nov. (CPAP-5007, holotype). a-b, 3D-rendering of partial left dentary with p2-p3 and m1-m3 in occlusal (a) and transparent dentary with teeth in ventral view (b). c, Tomographic slice showing the extra-root in p3 and the cross-section of the roots. d, 3D-rendering of dentary with teeth removed. Abbreviation: er, extra-root; rcr, radicular canal of the root.


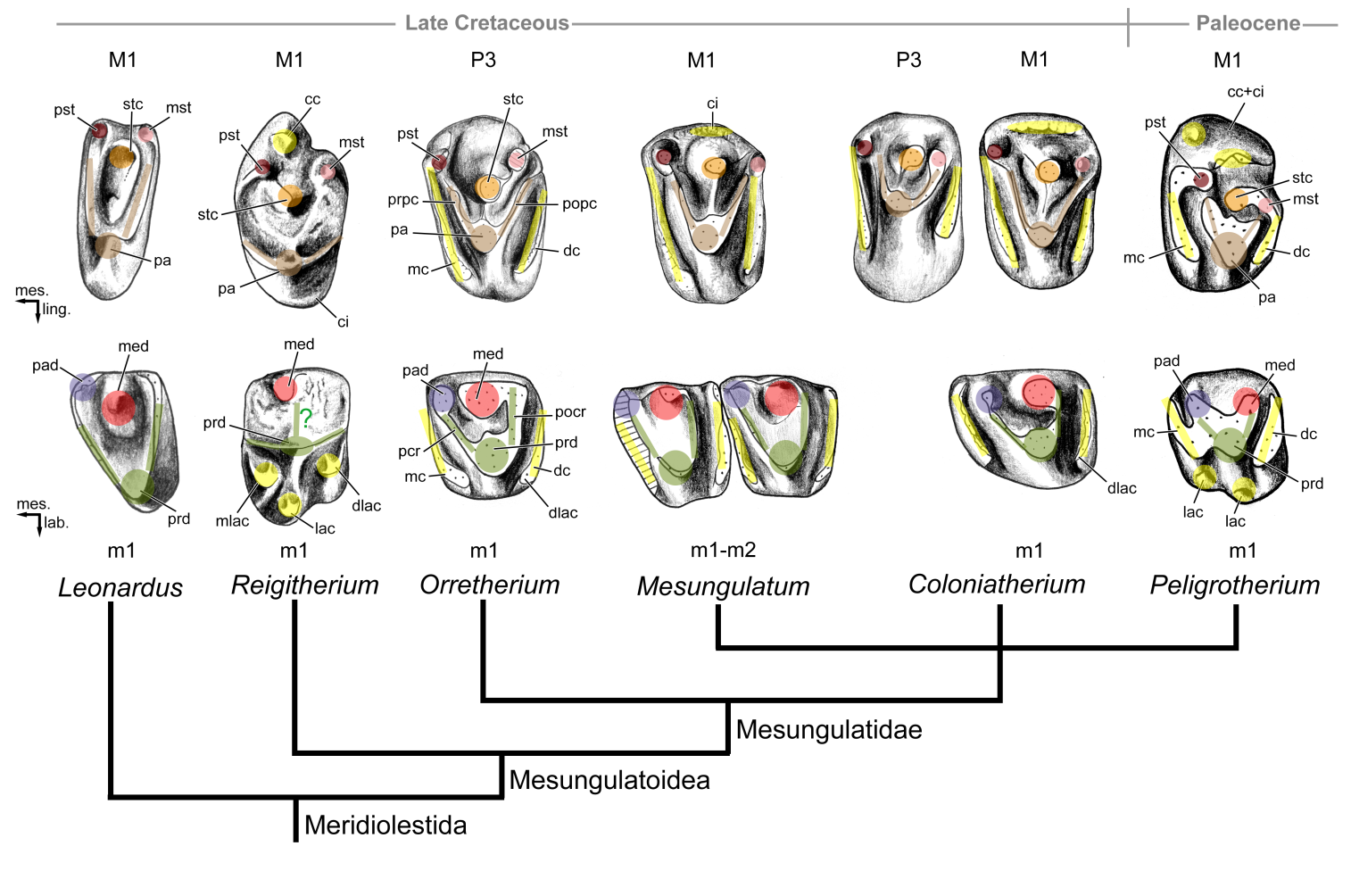


**Figure S3.** Comparison of selected left upper/lower postcanine teeth of some meridiolestian mammals, highlighting homologous structures with colours. *Leonardus* *cuspidatus* is based on MACN-RN 172 (M1) and MACN-RN 1097 (m1), tooth orientation as was interpreted by Bonaparte (1990) and Rougier et al., (2021); *Reigitherium bunodontum*, positions as inferred by Harper et al. (2019: fig. 3a and MPEF-PV 2238, M1; MPEF-PV 2317, m1); *Orretherium tzen*, CPAP-5008 (P3) and CPAP-5007 (m1); *Mesungulatum houssayi*, MACN-RN 03 (M1) and MACN-RN 06 (m1-m2); *Coloniatherium*, MPEF-PV 2081 (P3), MPEF-PV 2078 (M1) and MPEF-PV 2064 (m1), as interpreted by Rougier et al., (2009, 2021); and *Peligrotherium*, MPEF-PV 2351, as interpreted by Páez Arango (2008) and Rougier et al., (2021). For comparative purposes the M1/m1 of *Leonardus*, m1 of *Reigitherium*, P3 of *Reigitherium*, and P3 of *Coloniatherium* are inverted. Abbreviations: cc, cingular cusp; ci, cingulum; dc, distal cingulum; dlac, distolabial cingular cusp; mc, mesial cingulum; med, metaconid; mlac, mesiolabial cingular cusp; lac, labial cingular cusp; mst, metastyle; pa, paracone; pad, paraconid; pcr, preprotocristid; pst, parastyle; pocr, postprotocristid; popc, postparacrista; prd, protoconid; prpc, preparacrista; stc, stylocone.

**2. Tooth measurements of specimens of *Orretherium* *tzen* gen. et sp. nov.**

| **Specimen** | **Tooth** | **Mesio-distal (mm)** | **Labio-lingual (mm)** | **Dentary height, labial side (mm)** | **Dentary height, lingual side (mm)** |
| --- | --- | --- | --- | --- | --- |
| CPAP 5908 | P3 | 3.48 | 5.46 |  |  |
| CPAP 5907 | p2 | 4.01 | 3.32 |  |  |
|  | p3 | 3.78 | 3.97 | 4 | 5.58 |
|  | m1 | 3.12 | 3.64 |  |  |
|  | m2 | 3.53 | 3.98 |  |  |
|  | m3 | 3.03 | 3.6 | 4.72 | 6.35 |

**3. Tooth measurements of m1 of *Orretherium tzen* gen. et sp. nov. compared to other meridiolestidans. (*) estimated.**

|  |  | **Mesio-distal Length (mm)** | **Labio-lingual Width (mm)** | **Sources** |
| --- | --- | --- | --- | --- |
| *Necrolestes patagonensis* | | 1.1 | 1.54 | Wible and Rougier (2017) |
| *Reigitherium* *bunodontum* | | 1.4 | 1.9 | Harper et al. (2019) |
| *Leonardus cuspidatus* | | 1.47 | 2.25 | MACN-RN 172 |
| *Orretherium tzen* | | 3.12 | 3.64 | CPAP 5907 |
| *Mesungulatum houssayi* | | 3.9* | 3.7 | MACN-RN 06 |
| *Coloniatherium cilinskii* | | 5.7 | 4.85 | Rougier et al. (2021), MPEF-PV 2064 |
| *Peligrotherium tropicalis* | | 7.5 | 8.7 | Rougier et al. (2021), MPEF-PV 2351 |

**Table S1.** Measurements in m1 of selected meridiolestidan mammals.


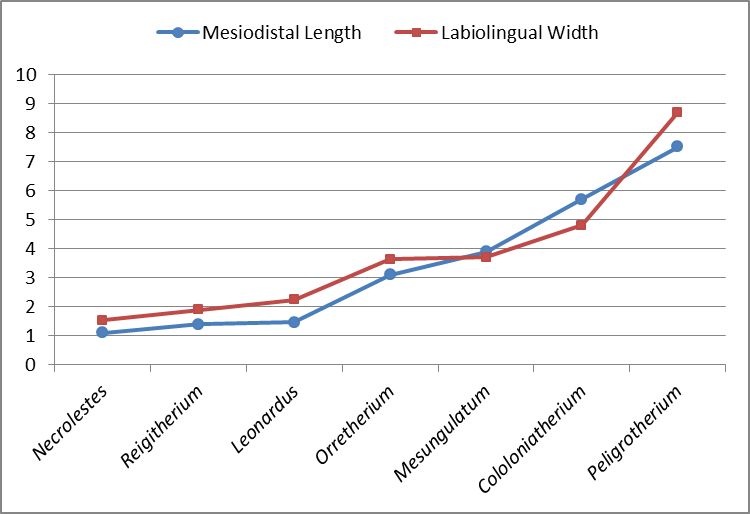


**Figure S4.** Plot of measurements (in mm) of m1 of selected meridiolestidan mammals.

**4. List of modifications to the data matrix of Rougier et al. (2012).**

The modifications made to the data matrix of Rougier et al. (2012) are marked with an asterisk **(*)**, and the characters and scoring changes provided by Harper et al. (2019) are marked with **(H)**. The data matrix also includes all the modifications of scorings in *Necrolestes* *patagonensis* provided by Wible and Rougier (2017, p. 249-251).

**Character 02** – *Peligrotherium* changes from 0 (Position of posteriormost mental foramen–Below the canine and anterior premolariform region) to 1 (Below the penultimate premolar). **(*) Based on Páez Arango (2008).**

**Character 05** – *Mesungulatum* changes from ? to 2 (Meckel’s groove in adults–Vestigial or absent). **(H)**

**Character 17** – *Reigitherium* changes from 1 (Ventral border of masseteric fossa–Present as a low and broad crest) to ?; *Peligrotherium* and *Coloniatherium* change from 1 to 0 (Absent); *Henkelotherium*, *Dryolestes* and *Laolestes* change from 2 (Present as a well-defined and thin crest) to 1. **(H)**

**Character 18** – *Reigitherium* changes from ? to 1 (Position of mandibular foramen–Posterior to the anterior edge of the coronoid process). **(H)**

**Character 29** – *Cronopio*, *Coloniatherium* and *Peligrotherium* change from 0 (Ultimate functional molar is medial to the coronoid process) to 1 (Ultimate functional molar is in alignment with the anterior margin of coronoid process). **(*)**

**Character 40** – *Reigitherium* changes from 1/2 (Total number of premolars–Three/four) to 2 (four). **(H)**

**Character 42** – Harper et al. (2019) changed from ? to 2 in *Mesungulatum*. (*) **We not followed it and kept the original scoring ?, because this premolar position is unkwown for *Mesungulatum*.**

**Character 45** – *Peligrotherium* changes from 1 (Paraconid, last lower premolar–Small, much smaller than cusp c, or vestigial to absent) to 0 (Present, at least subequal to cusp c, or posterior cingular cusp of the same tooth). **(*) Based on specimen MPEF-PV 2351 (Páez Arango, 2008; Rougier et al., 2021).**

**Character 48** – *Reigitherium* changes from 1 (Last lower premolar outline–Transversely wide, crown outline subequal or wider than long) to 0 (Laterally compressed, crown outline longer than wide). **(*) The p4 of *Reigitherium* (see Harper et al., 2019) is mesio-distally longer than transversely wide, narrower than first molar; it is different to the condition of the last premolar of *Coloniatherium*, *Peligrotherium* (Rougier et al., 2009, 2021; Páez Arango, 2008), and *Orretherium*.**

**Character 49** – Harper et al. (2019) changed in *Mesungulatum* from ? to 2 (Last lower premolar size–Hypertrophied, much larger than any molar). **We did not follow it and kept the original scoring ?, because this tooth position is unknown in this taxon.**

**Character 51** – *Mesungulatum* changes from 1 (Lower premolars lingual cingulid–Present) to ?. **(*) These tooth positions are unknown in this taxon.**

**Character 53** – *Reigitherium* changes from 0 (Relative height of primary cusp a to c of last lower premolar,–Posterior cusp c is absent or very small) to 1 (Posterior cusp c is distinctive but less than 30% of primary cusp a). **(*) The metaconid (cusp c) in the last premolar (p4) of *Reigitherium* is present and evident (see Harper et al., 2019).**

**Character 55** – *Reigitherium* changes from 1/2 (Number of molars-lowers preferred–Three/four-five) to 1 (three). **(H)**

**Character 63** – *Reigitherium* changes from 0 (Relative elevation of bases of paraconid (cusp b) and metaconid (cusp c)–Almost or at the same level) to ?. **(*) In Ch. 62, the relative size and height of paraconid to metaconid was previously scored as ? and as stated by Harper et al. (2019) the paraconid is very small/absent in *Reigitherium*, thus we made this change in Ch. 63 that is concomitant with the score in Ch. 62.**

**Character 78** – *Reigitherium* changes from 1 (Orientation of paracristid relative to longitudinal axis of molars–Oblique) to 2 (Nearly transverse). **(H)**

**Character 79** – *Reigitherium* changes from 0 (Paraconid on lower molars–Present) to 1 (Absent). **(H)**

**Character 80** – *Reigitherium* changes from 0 (Mesiolingual surface of paraconid on lower molars, rounded) to ?. **(*) Based on Harper et al. (2019), the paraconid is absent in *Reigitherium* (see Ch. 79).**

**Character 81** – *Reigitherium* changes from 0 (Procumbent paraconid–Absent) to ?. **(*) Based on Harper et al. (2019) on absence of paraconid (see Ch. 79).**

**Character 82** – *Reigitherium* changes from 2 (Proximity between paraconid and metaconid–Single cusp (amphyconid)) to ?. **(*) Based on the morphology of p4 of *Reigitherium* (see Harper et al., 2019), the absence of paraconid in molars (as m1) cannot certainly assumed as it is appressed onto the metaconid (i.e., amphiconid), which is far distant from its position (although very small) in p4.**

**Character 106** – *Reigitherium* changes from 0 (Central crest in triangular upper molariforms–Absent) to 0/1 (Absent/present). **(H)**

**Character 108** – Harper et al., (2019) changed in *Reigitherium* and *Peligrotherium* from 1 (crown length/width ratio among lower molariforms–Subequal) to 2 (Crown wider than long). **(*) The change is not followed for *Peligrotherium*; we kept stage 1, in which lower molars are subequal in crown Length/Width ratio (see** **Páez Arango, 2008; Rougier et al., 2021), similar to the condition seen in *Coloniatherium* and *Mesungulatum*.**

**Character 110** – *Mesungulatum* and *Coloniatherium* change from 2 (Aspect ratio of M1– Triangular outline) to 4 (Rectangular or nearly so). **(*) Based on descriptions provided by Harper et al. (2019) and Rougier et al. (2021).**

**Character 120** – *Reigitherium* changes from 1 (Metacristid (protocristid) orientation on posterior molariforms–Oblique) to 2 (Transverse); *Peligrotherium* changes from 1/2 to 2; *Mesungulatum* changes from 1 to 2. **(H)**

*Coloniatherium* changes from 1 to 2. **(*) Based on tooth reconstruction presented in Rougier et al. (2021).**

**Character 122** – *Mesungulatum* changes from 1 (Bifid metastyle–Present) to 0 (absent). **(*) It is not seen in *Mesungulatum houssayi* (Bonaparte, 1986).**

**Character 136** – *Reigitherium* changes from 0 (Lower molar roots–Subequal) to 1 (Posterior molar root much smaller). **(H)**

**Character 140** – *Reigitherium* changes from ? to 0 (Trigon major axis orientation–Labially). **(H)**

**Character 142** – *Reigitherium* changes from 1 (Precingulum upper molar,

developed forming a lingual cusp) to 0 (Narrow and closely attached to crown, to absent). **(*) A precingulum is not developed in upper molars of *Reigitherium* (see Harper et al., 2019; Rougier et al., 2021), showing a conspicuous difference from the condition of *Mesungulatum*, *Coloniatherium*, and *Peligrotherium*. See Fig. S3.**

**Character 143** – *Reigitherium* changes from 1 (Postcingulum upper molar,

developed forming a lingual cusp) to 0 (Narrow and closely attached to crown, to absent). **(*) A postcingulum is not developed in upper molars of *Reigitherium* (see Harper et al., 2019; Rougier et al., 2021), showing a conspicuous difference from the condition of *Mesungulatum*, *Coloniatherium*, and *Peligrotherium*. See Fig. S3.**

**Character 144** – *Reigitherium* changes from 2 (Cingula pre- and postcingula/cingulid height–elevated reaching occlusal surface) to 0 (Absent or little differentiated). **(*) A pre- and postcingulum is not developed in upper molars of *Reigitherium* (see Harper et al., 2019; Rougier et al., 2021), showing a conspicuous difference from the condition of *Coloniatherium*, *Mesungulatum*, and *Peligrotherium*. See Fig. S3.**

**Character 149** – *Reigitherium* changes ? to 0 (Supernumerary roots on penultimate lower premolar–Absent). **(*) Based on the description provided by Harper et al. (2019).**

**Character 150** – *Reigitherium* changes from 1 (Supernumerary roots on ultimate lower premolar–Present) to ?. **(H)**

**(*) Based on the description provided by Harper et al. (2019) we score this character as (Supernumerary roots on ultimate lower premolar, Absent).**

**Character 151** – *Reigitherium* changes from 1 (Supernumerary roots on ultimate upper premolar–Present) to ?. **(H)**

**Character 152** – *Reigitherium* changes from ? to 0 (Penultimate lower premolar distal root–Subequal to mesial root). **(H)**

**Character 153** – Harper et al. (2019) changed in *Reigitherium* from 0 (Lower molars contact each other somewhere along the mesial and distal edges of the crown) to ?. **We did not follow this change and maintained the original scoring 0, as inferred by its tooth row reconstruction (see Harper et al., 2019 and Rougier et al., 2021).**

**Character 154** – *Reigitherium* changes from 0 (Upper molars extensively contact each other) to ?. **(H)**

**5. Character list used in the phylogenetic analysis.**

This list is taken from Rougier et al. (2012) and characters 318 to 321 were taken from Harper et al. (2019).

**1 - Symphysis Height**: 0. Robust, verticalized; 1. Slender, oblique, or approaching horizontal.

**2 - Position of Posteriormost Mental Foramen**: 0. Below the canine and anterior premolariform region; 1. Below the penultimate premolar; 2. Below the ultimate premolar; 3. Between the ultimate premolar and the first molar.

**3 - Postdentary Trough. Behind tooth row**: 0. Present; 1. Absent.

**4 - Mandibular Alveolar Border**: 0. Subequal in height to the lingual edge; 1. Labial border much lower than lingual.

**5 - Degree of Development of Meckelian Groove in Adults**: 0. Well developed; 1. Weakly developed; 2. Vestigial or absent.

**6 - Curvature of Meckelian Groove in Adults. Under the tooth row**: 0. Parallel to the ventral border of mandible; 1. Convengent to the ventral border of mandible.

**7 - Groove for Replacement Dental Lamina**: 0. Present; 1. Absent.

**8 - Angular Process Presence**: 0. Absent; 1. Present.

**9 - Angular Process Direction**: 0. Small knobby process, not projected; 1. Straight process, posteriorly directed; 2. Transversely flaring; 3. Inflected; 4. Downturned.

**10 - Antero-Posterior Position of Angular Process Relative to Dentary Condyle**: 0. Anterior position, the angular process is below the main body of the coronoid process; 1. Posterior position, the angular process is placed at the level of the posterior end of the coronoid process.

**11 - Vertical Position of Angular Process**: 0. Low, at or near the level of the ventral border of the mandibular horizontal ramus; 1. High, at or near the level of the molar alveolar line.

**12 - Coronoid, or Its Attachment Scar, in Adults**: 0. Present; 1. Absent.

**13 - Medial Fossa on Dentary Angular Process**: 0. Present; 1. Absent.

**14 - Pterygoid Fossa**: 0. Absent; 1. Present.

**15 - Medial Pterygoid Ridge or Shelf Direction**: 0. Directed to angular process; 1. Reaching the dentary condyle via a low crest.

**16 - Pterygoid Shelf**: 0. Absent; 1. Present.

**17 - Ventral Border of Masseteric Fossa**: 0. Absent; 1. Present as a low and broad crest; 2. Present as a well-defined and thin crest.

**18 - Position of Mandibular Foramen**: 0. Below or near to the base of the anterior border of the coronoid process; 1. Posterior to the anterior edge of the coronoid process.

**19 - Masseteric Foramen**: 0. Absent; 1. Present.

**20 - Crest of Masseteric Fossa Along Anterior Border of Coronoid Process**: 0. Absent or weakly developed; 1. Present as a distinct anterior border.

**21 - Mylohyoid Process at Level of Anterior Border of Coronoid Process**: 0. Absent; 1. Present.

**22 - Orientation of Dentary Peduncle and Condyle**: 0. Dentary peduncle is posteriorly directed, forms an angle of 40° or less to the alveolar margin; 1. Vertically directed dentary peduncle, above 40°; 2. Dentary condyle is continuous with the semicircular posterior margin of the dentary.

**23 - Lower Mandibular Margin/Condylar Peduncle**: 0. Not continuous, interrupted by an angular process or a sharp angle; 1. Continuous as a single line in lateral view.

**24 - Shape and Relative Size of Dentary Articulation**: 0. Small and dorsoventrally compressed; 1. Condyle is massive and bulbous, transversely broad in its dorsal aspect; 2. Condyle mediolaterally narrow and vertically deep, forming a broad arc in lateral outline, either ovoid or triangular in posterior view.

**25 - Ventral Border of Dentary Peduncle**: 0. Posteriorly tapering without a condyle; 1. Columnar or ridge-like; 2. Ventrally flaring; 3. Robust and short.

**26 - Position of Dentary Condyle Relative to Vertical Level of Postcanine Alveoli**: 0. Below or about the same level as the postcanine alveoli; 1. Above the level of the postcanine alveoli.

**27 - Tilting of Coronoid Process of Dentary. Measured as the angle between the imaginary line of the anterior border of the coronoid process and the horizontal alveolar line of all molars**: 0. Coronoid process is strongly reclined forming an obtuse angle; 1. Coronoid process less reclined, 135–145°; 2. Coronoid process less than vertical, 115–125°; 3. Coronoid process is near vertical and the angle is small, 95–105°.

**28 - Retromolar Space. At least half the length of the last molar**: 0. Absent; 1. Present.

**29 - Alignment of Ultimate Molar to Anterior Margin of Dentary Coronoid Process**: 0. Ultimate functional molar is medial to the coronoid process; 1. Ultimate functional molar is in alignment with the anterior margin of coronoid process.

**30 - Shape of Ventral Edge of Jaw in Area of Angular Process**: 0. Straight; 1. Convex; 2. Concave.

**31 - Incisor Replacement**: 0. Alternating and multiple replacement; 1. Diphyodont replacement or none.

**32 - Number of Lower Incisors**: 0. Three or more; 1. Two or fewer.

**33 - Maxillary Incisor**: 0. Present; 1. Absent.

**34 - Staggered Incisor**: 0. Absent; 1. Present.

**35 - Canine Replacement**: 0. Multiple replacements; 1. Diphyodont.

**36 - Canines**: 0. Present and large; 1. Present and small; 2. Absent.

**37 - Upper Canine Height**: 0. Long, at least twice the height of tallest postcanine; 1. Short, less than twice the height of the tallest postcanine.

**38 - Long Upper Canine Height**: 0. Relatively short, less than three times the height of the tallest postcanine crown; 1. Tall, more than three times the height of tallest postcanine crown.

**39 - Replacement of Premolariforms**: 0. Multiple replacement; 1. One replacement or none.

**40 - Total Number of Premolars. Lower premolar preferred; uppers used if no lowers are available**: 0. Two or fewer; 1. Three premolars; 2. Four premolars; 3. Five or more.

**41 - Diastema Separating P1 From P2. Distance equal or larger than half of the P1**: 0. Absent; 1. Present.

**42 - Penultimate Lower Premolar Size**: 0. Small and subequal to other premolars; 1. Larger than any other premolar, longer and/or taller; 2. Hypertrophied, dominant tooth in the series.

**43 - Penultimate Lower Premolar—Paraconid (Cusp b)**: 0. Much smaller than metaconid (cusp c) of the same tooth, or absent; 1. Well developed as an important cusp of trigonid.

**44 - Last Lower Premolar—Symmetry of Main Cusp a (Protoconid)**: 0. Asymmetrical, anterior edge of cusp a is more convex in outline than the posterior edge; 1. Symmetrical, anterior and posterior cutting edges are equal or subequal in length.

**45 - Last Lower Premolar—Anterior Cusp b (Paraconid)**: 0. Present, at least subequal to cusp c, or posterior cingular cusp of the same tooth; 1. Small, much smaller than cusp c or posterior cingular cusp of the same tooth, or vestigial to absent.

**46 - Last Lower Premolar—Arrangement of Principal Cusp a, Cusp b (if Present), and Cusp c**: 0. Aligned straight or at a slight angle; 1. Distinctive triangulation.

**47 - Last Lower Premolar—Distinct Distal Cingulid Cusp d**: 0. Absent; 1. Posterior cingular cusp present; 2. Present as part of a continuous distal cingulid.

**48 - Last Lower Premolar Outline**: 0. Laterally compressed, crown outline longer than wide; 1. Transversely wide, crown outline subequal or wider than long.

**49 - Last Lower Premolar Size**: 0. Small and subequal to other premolars; 1. Large tooth, taller than or subequal to first molar; 2. Hypertrophied, much larger than any molar.

**50 - Labial Cingulid of Last Lower Premolar**: 0. Absent or vestigial; 1. Present along more than half of the crown length.

**51 - Lower Premolars Lingual Cingulid**: 0. Absent or vestigial; 1. Present.

**52 - Relative Height of Primary Cusp a to c of Last Lower Premolar. Measured as the height ratio of a and c from the bottom of the valley between the two adjacent cusps**: 0. Posterior cusp c is absent or very small; 1. Posterior cusp c is distinctive but less than 30% of primary cusp a; 2. Posterior cusp c and primary cusp a are equal or subequal in height (c is 40–100% of a).

**53 - Last Upper Premolar—Functional Protocone**: 0. Absent; 1. Present.

**54 - Last Upper Premolar—Parastylar Hook**: 0. Present; 1. Absent or very small.

**55 - Number of Molars or Molariform Postcanines. Lowers preferred, uppers used if no lowers are known**: 0. Two molars; 1. Three molars; 2. Four to five molars; 3. Six or more.

**56 - Alignment of Main Cusps of Posterior Lower Molars. m3 or more posterior teeth considered if present**: 0. Simple longitudinal row; 1. Obtuse angle (>95); 2. Acute angle (<90).

**57 - Alignment of Main Cusps of Anterior Lower Molar (m1)**: 0. Single longitudinal row; 1. Acute angle; 2. Obtuse angle.

**58 - Development of Postprotocrista on Upper Molars for Double Rank Postvallum Shear. For molars with reversed triangulation of molarcusps**: 0. Postprotocrista is short and does not extend labially beyond metacone; 1. Postprotocrista is long and extends labially beyond metacone.

**59 - Precise Opposition of Upper and Lower Molars. Either one-to-one, or occluding at the opposing embrasure or talonid**: 0. Absence of precise opposition of upper and lower molars; 1. Present (either one-to-one or occluding at the opposing embrasure or talonid).

**60 - Relationships Between Cusps of Opposing Upper and Lower Molars**: 0. Absent; 1. Present, lower primary cusp a occludes in the groove between upper cusp a and b; 2. Present, lower main cusp a occludes in front of upper cusp b and into the embrasure between the opposing and preceding upper teeth; 3. Present, part of the talonid occluding with the lingual face (or any part) of the upper molar; 4. Lower multicuspate rows alternately occlude between the upper multicuspate rows.

**61 - Relative Height of Primary Cusp a (Protoconid) to Cusp c (Metaconid) of Anterior Lower Molars. Measured as the height ratio of a and c from the bottom of the valley between the two adjacent cusp, on m1**: 0. Posterior cusp c is less than 40% of the primary cusp a (protoconid); 1. Posterior cusp c is more than 40% of cusp a.

**62 - Relative Size/Height of Cusp b (Paraconid) to Cusp c (Metaconid). Based on m2 when possible**: 0. c taller than b; 1. b and c subequal in height; 2. b taller than c.

**63 - Relative Elevation of Bases of Paraconid (Cusp b) and Metaconid (Cusp c)**: 0. Almost or at the same level; 1. Base of the paraconid higher than base of the metaconid; 2. Base of metaconid higher than base of the paraconid.

**64 - Cristid Obliqua**: 0. Absent; 1. Present.

**65 - Cristid Obliqua Orientation**: 0. Oriented to or lingual to the metaconid-protoconid notch; 1. Hypertrophied and directed to posterior part of the metaconid; 2. Short and pointed anteriorly between the metaconid-protoconid notch and the protoconid.

**66 - Lower Molar—Pre-Entocristid on Talonid Heel**: 0. Talonid lacks medial and longitudinal crest; 1. Pre-entoconid cristid of talonid in alignment with the metaconid or with the postmetacristid if the latter is present; 2. Pre-entocristid crest is offset from the metaconid and it is lingual to the base of the metaconid.

**67 - Labial Curvature of Primary Cusp a of Lower Molars (at Base Level) Relative to Curvature of Cusps b and c**: 0. Cusps a, b, and c have the same degree of bulging; 1. Cusp a is far more bulging than cusps b and c.

**68 - Labial Curvature of Main Cusps a, b, and c at Level of Cusp Valley of Penultimate and Ultimate Upper Molars**: 0. Cusp a, b, and c have about the same degree of curvature; 1. Cusp a is slightly concave (or far less convex than either cusp b or c).

**69 - Labiolingual Compression of Primary Functional Cusps of Lower Molars. At the level of the cusp base but above the cingulid**: 0. Absent; 1. Present.

**70 - Posterior Lingual Cingulid of Lower Molars**: 0. Absent or weak; 1. Distinctive; 2. Strongly developed, crenulated with distinctive cuspules.

**71 - Cingular Cuspule e on Lower Molars**: 0. Present; 1. Absent.

**72 - Cingular Cuspule f on Lower Molars**: 0. Absent; 1. Present.

**73 - Mesial Transverse Cingulid.** 0. Absent; 1. Present as a continuous shelf below the trigonid without occlusal function; 2. Present, a continuous shelf below, having occlusal contact with the upper molar.

**74 - Cingulid Shelf Wrapping Around Anterointernal Corner of Lower Molar to Extend to Lingual Side of Trigonid Below Paraconid**: 0. Absent; 1. Present, weakly developed restricted to the mesial aspect of the paraconid base; 2. Present, strongly developed, running along most of the lingual base of the paraconid.

**75 - Postcingulid**: 0. Absent; 1. Present, oblique, and connected to hypoconulid; 2. Present, continue and horizontal above the gum level; 3. Present as a distolabial cingulum, above the gum level.

**76 - Interlocking Mechanism Between Two Adjacent Lower Molars**: 0. Absent; 1. Present, posterior cingular cuspule d of the preceding molar fits in between cingular cuspules e and f of the succeeding molar or flat surfaces of mesial cingula or cusp b; 2. Present, posterior cingular cuspule d fits between cingular cuspule e and cusp b of the succeeding molar; 3. Present, posterior cingular cuspule d of the preceding molar fits into and embayment or vertical groove of the anterior aspect of cusp b the succeeding molar.

**77 - Size Ratio of Posterior Molars. Lower molar preferred when available**: 0. Last three postcanines forming a series of posteriorly decreasing size; 1. Penultimate molar is the largest of molars; 2. Ultimate molar is the largest of molars.

**78 - Orientation of Paracristid Relative to Longitudinal Axis of Molars**: 0. Longitudinal orientation; 1. Oblique; 2. Nearly transverse.

**79 - Paraconid Presence on Lower Molars**: 0. Present; 1. Absent.

**80 - Mesiolingual Surface of Paraconid on Lower Molars**: 0. Rounded; 1. Forming a keel.

**81 - Procumbent Paraconid on Lower Molars**: 0. Absent; 1. Present, projected as a conical cusp beyond crown base.

**82 - Proximity Between Paraconid and Metaconid**: 0. Bases widely separated; 1. Bases approaching each other becoming confluent; 2. Single cusp (amphyconid).

**83 - Molar Trigonid/Talonid Width Ratio**: 0. Narrow or absent (talonid <40% of trigonid); 1. Wide (talonid is 40–70% of the trigonid); 2. Talonid is equal or wider than trigonid (above 70% of the width of the trigonid).

**84 - Lower Molar Hypoflexid**: 0. Absent or shallow; 1. Deep (but less than 50% of the talonid width); 2. Very deep (>60% of the talonid width).

**85 - Talonid Basin**: 0. Absent; 1. Present.

**86 - Morphology of Rear Portion of Molariform**: 0. Single cusp (d), cingulum or absent; 1. Present as an incipient heel; 2. Present as a heel (with at least one functional cusp); 3. Present as a transverse V-shaped basin with two major cusps; 4. Rimmed with three major cusps.

**87 - Hypoconulid (=Cusp d)**: 0. Hypoconulid at the cingulid level; 1. Hypoconulid elevated above the cingulid level.

**88 - Hypoconid**: 0. Absent; 1. Present.

**89 - Hypoconulid Orientation**: 0. Cusp tip erect or procumbent; 1. Cusp tip recumbent (reclined posteriorly).

**90 - Entoconid**: 0. Absent; 1. Present but far from hypoconulid, at least equal to one cusp length; 2. Present and twinned with hypoconulid.

**91 - Height of Entoconid Compared With Other Talonid Cusps**: 0. Lower than the hypoconulid (or even vestigial); 1. Subequal height to the hypoconulid.

**92 - Alignment of Paraconid, Metaconid, and Entoconid**: 0. Cusps not aligned; 1. Cusps aligned.

**93 - Aspect Radio in Occlusal View (Length vs. Width) of Functional Talonid Basin at Cingulid Level**: 0. Longer than wide; 1. Length equals width; 2. Wider than long.

**94 - Elevation of Talonid**: 0. Hypoconulid/protoconid height ratio less than 20% (hypoconulid = cusp d); 1. Hypoconulid/protoconid height ratio between 25% and 35%; 2. Hypoconulid/protoconid height ratio between 40% and 50%; 3. Hypoconulid/protoconid height ratio: 50% or higher.

**95 - Width of Upper Molar Stylar Shelf**: 0. Present and broad; 1. Present and narrow; 2. Absent.

**96 - Labial Cingulum of Upper Molars**: 0. Absent or weak; 1. Distinctive cingulum, straight; 2. Distinctive cingulum with strong ectoflexus (but without hypertrophied stylar cusps); 3. Wide cingulum with distinctive ectoflexus, plus individualized and hypertrophied stylar cusps; 4. Cingulum with distinctive and even-sized multiple cuspules.

**97 - Upper Molars With Functional Lingual Protocone That Grinds Against Basin on Lowers**: 0. Absent; 1. Present.

**98 - Trigon Basin. Protocone must be present**: 0. Absent; 1. Present, the labial slope of the protocone determines a basin against the lingual slope of the paracone/metacone.

**99 - Transverse Width of Protocone on Upper Molars. Protocone must be present**: 0. Narrow (distance from the protocone apex to paracone apex; <0.60 of total tooth width); 1. Strongly transverse (distance from the protocone apex >0.60 of total width).

**100 - Anteroposterior Development of Lingual Region on Upper Molars. Protocone must be present**: 0. Narrow (anteroposterior distance medial to the paracone; and metacone <0.30 of total tooth length); 1. Moderate development (distance between position of conules = 0.31–0.50 of total tooth length); 2. Long (distance between conules >0.51 of total tooth length).

**101 - Conules on Upper Molars**: 0. Absent; 1. Present but weak and without cristae; 2. Conules distinctive, with cristae.

**102 - Relative Height and Size of Paracone (Cusp b) and Metacone of Upper Molars**: 0. Paracone higher and larger than metacone; 1. Metacone is higher and larger than paracone.

**103 - Centrocrista Between Paracone and Metacone of Upper Molars. Consider only for tribosphenic forms**: 0. Straight; 1. V-shaped, with labially directed postparacista and premetacrista.

**104 - Upper Molars Cuspule e**: 0. Present; 1. Absent.

**105 - Upper Molar Interlock**: 0. Absent; 1. Tongue-in-groove interlock.

**106 - Central Crest (Medianergrat) in Triangular Upper Molariforms**: 0. Absent; 1. Present.

**107 - Outline of m1**: 0. Oval-shaped; 1. Laterally compressed; 2. Oblong with slight labial bulge; 3. Oblong with strong labial bulge; 4. Triangular or tear-drop shaped; 5. Rectangular or slightly rhomboidal.

**108 - Crown Length/Width Ratio Among Lower Molariforms**: 0. Crown longer than wide; 1. Crown length/width subequal; 2. Crown wider than long.

**109 - Shape of Lingual Margin in Lower Molars**: 0. Notched; 1. Flat.

**110 - Aspect Ratio of M1**: 0. Laterally compressed; 1. Oval-shaped or spindle-shaped; 2. Triangular outline; 3. Dumbbell-shaped; 4. Rectangular or nearly so.

**111 - Crown Height Difference Between Buccal and Lingual Sides in Lower Molariforms**: 0. Buccal and lingual sides of similar height; 1. Buccal side much taller than lingual side.

**112 - Functional Development of Occlusal Facets on Individual Molar Cusps**: 0. Absent for lifetime; 1. Absent at eruption but development later by extensive wearing of the crown; 2. Wear facet develops on a morphology approximately present upon eruption.

**113 - Topographic Relationships of Wear Facets to Main Cusps**: 0. Lower cusps a and c support two different wear facet (1 and 4) that contact the upper main cusp a; 1. Lower cusps a and c support a single wear facet (4) that contacts the upper primary cusp b.

**114 - Development and Orientation of Prevallum/Postvallid Shearing**: 0. Absent; 1. Present and obtuse; 2. Present, hypertrophied, and transverse.

**115 - Upper Molar—Development of Facet 1 and Preprotocrista (or Paracrista) on Upper Molars**: 0. Facet 1 (prevallum crest) is short, and does not extend to the stylocone area; 1. Wear facet 1 extends beyond into the hook-like area near the stylocone; 2. Long preprotocrista (below the paracone-stylocone crest) is added to the prevallum shear and extends labially beyond paracone.

**116 - Differentiation of Wear Facet 3 and Wear Facet 4**: 0. Absent; 1. Present; 2. Facet hypertrophied on the flanks of the strongly V-shaped talonid.

**117 - Orientation of Wear Facet 4**: 0. Present and oblique to long axis of the tooth; 1. Present and forming a more transverse angle to long axis of the toot.

**118 - Wear Pattern on Talonid**: 0. Absent; 1. Present.

**119 - Direction of Jaw Movement During Occlusion**: 0. Dorsomedial movement; 1. Dorsomedial movement with a significant medial component; 2. Dorsoposterior movement.

**120 - Metacristid (Protocristid) Orientation on Posterior Molariforms**: 0. Parallel to lower jaw axis; 1. Oblique; 2. Transverse.

**121 - Bifid Metaconid**: 0. Absent; 1. Present.

**122 - Bifid Metastyle**: 0. Absent; 1. Present.

**123 - Distal Metacristid**: 0. Present; 1. Absent.

**124 - Superficial Features on Occluding Surface of Wear Facets 5 and 6 in Talonid for Basined Talonid**: 0. Smooth surface on the talonid (or on cusp d); 1. Multiple ridges within talonid basin.

**125 - Position of Stylocone in Posterior Molariforms**: 0. Along buccal edge; 1. Separated

**126 - Stylocone Relationship in Triangular Teeth**: 0. Stylocone connected to paracrista or mesial to its end; 1. Stylocone distal to labial ending of paracrista; 2. Stylocone detached of preparacrista occupying central position on crown.

**127 - Stylocone Size in Triangular Teeth**: 0. Absent; 1. Small stylar cusp; 2. Prominent cusp subequal or larger than paracone.

**128 - Parastylar Hook in Upper Molars**: 0. Absent or poorly developed; 1. Present.

**129 - Paracone Orientation**: 0. Erect; 1. Recumbent; 2. Procumbent.

**130 - Metacone**: 0. Present; 1. Absent.

**131 - Paracone-Metacone Labiolingually Aligned. Consider only in subtriangular upper teeth**: 0. Absent, the metacone is labial to paracone; 1. Present, the metacone is aproximately aligned mesiodistally with the paracone.

**132 - Accessory Cusps on Bucal Side of Upper Molars**: 0. Absent; 1. Present.

**133 - Deep Ectoflexus on Upper Molars**: 0. Present only on penultimate/ultimate molar; 1. On penultimate and preceding molar; 2. Strongly reduced to absent.

**134 - Lower Molariform Roots**: 0. Incipient or incomplete separation; 1. Root divided.

**135 - Number of Lower Molariform Roots**: 0. One; 1. Two; 2. Three or more.

**136 - Size of Lower Molar Roots**: 0. Subequal; 1. Posterior molar root much smaller; 2. Single root (posterior root absent); 3. Anterior root smaller.

**137 - Lower Molar Root Cross Section. m2 considered when available**: 0. Circular/subcircular; 1. Anteroposteriorly compressed.

**138 - Replacement of at Least Some Functional Molariforms**: 0. Present; 1. Absent.

**139 - Procumbent and Enlargement of Anteriormost Lower Incisor**: 0. Absent; 1. Present, both procumbent and enlarged more than 50% the second.

**140 - Trigon Major Axis Orientation. As indicated by the distal wall of the trigonid**: 0. Labially; 1. Mesially; 2. Sharply distal.

**141 - Lingual Cingulum/Trigon on Paracone. Trigon and/or protocone are considered elaborations of the cingulum**: 0. Absent; 1. Present.

**142 - Precingulum upper molar**: 0. Narrow and closely attached to crown, to absent; 1. Developed forming a lingual cusp.

**143 - Postcingulum**: 0. Narrow and closely oppresed to crown, to absent; 1. Developed forming a lingual cusp.

**144 - Cingula Pre- and Postcingula/Cingulid Height**: 0. Absent or little differentiated; 1. Close to crown base; 2. Elevated reaching occlusal surface.

**145 - Prehypoconulid Crest. A crest connecting the metaconid with the hypoconulid along the lingual edge of the tooth**: 0. Absent; 1. Present.

**146 - Number of Upper Molariform Roots**: 0. One; 1. Two; 2. Three; 3. More than three.

**147 - Position of Lingual Upper Root**: 0. Under paracone; 1. Under protocone or trigon.

**148 - Supernumerary Roots on Penultimate Upper Premolar**: 0. Absent; 1. Present.

**149 - Supernumerary Roots on Penultimate Lower Premolar**: 0. Absent; 1. Present

**150 - Supernumerary Roots on Ultimate Lower Premolar**: 0. Absent; 1. Present.

**151 - Supernumerary Roots on Ultimate Upper Premolar**: 0. Absent; 1. Present

**152 - Penultimate Lower Premolar Distal Root**: 0. Subequal to mesial root; 1. Large elongated root, more than 50% of crown length.

**153 - Lower Molar Contact**: 0. Lower molars contact each other somewhere along the mesial and distal edges of the crown; 1. Lower molars do not contact each other being separated by interdental spaces.

**154 - Upper Molar Contact**: 0. Upper molars extensively contact each other; 1. Upper molars do not contact each other, or barely do so.

**155 - Enamel Prism Shape**: 0. Prisms absent; 1. Arc; 2. Enclosed.

**156 - Enamel Prism Seams**: 0. Present; 1. Absent.

**157 - Enamel Prism Packing**: 0. Hexagonal; 1. Erratic; 2. In rows.

**158 - Fusion of Atlas Neural Arch and Intercentrum in Adults**: 0. Unfused; 1. Fused.

**159 - Atlas Ribs in Adults**: 0. Present; 1. Absent.

**160 - Fusion of Dens to Axis**: 0. Unfused; 1. Fused.

**161 - Rib of Axis in Adults**: 0. Free ribs present; 1. Ribs fuse to become transverse process.

**162 - Postaxial Cervical Ribs in Adult**: 0. Free ribs present; 1. Free ribs absent.

**163 - Thoracic Vertebrae**: 0. 13 thoracic vertebrae; 1. 15 or more thoracic vertebrae.

**164 - Lumbar Ribs**: 0. Unfused to vertebrae; 1. Synostosed to vertebrae to form transverse processes.

**165 - Interclavicle in Adults**: 0. Present; 1. Absent.

**166 - Contact Relationships in Adults Between Interclavicle and Sternal Manubrium**: 0. Posterior end of interclavicle abuts anterior border of manubrium; 1. Interclavicle broadly overlaps the ventral side of the manubrium; 2. Complete fusion of the embryonic membranous and endochondral elements.

**167 - Cranial Margin of Interclavicle**: 0. Anterior border is emarginated or flat; 1. With a median process (assuming interclavicle is fused to the sternal manubrium in living therians).

**168 - Claviculo-Sternal Apparatus Joint**: 0. Immobile; 1. Mobile.

**169 - Acromioclavicular Joint**: 0. Extensive articulation; 1. Limited articulation.

**170 - Curvature of Clavicle**: 0. Boomerang-shaped; 1. Slightly curved.

**171 - Scapula—Supraspinous Fossa**: 0. Absent; 1. Weakly developed, present only along a part of the scapula; 2. Fully developed and present along the entire dorsal border of scapula.

**172 - Scapula—Acromion Process**: 0. Short, (even with or behind the glenoid); 1. Hook-like and extending below the glenoid.

**173 - Scapula—Fossa or Process for Teres Major Muscle**: 0. Absent; 1. Present.

**174 - Procoracoid**: 0. Present as a free element; 1. Fused to the sternal apparatus in adult.

**175 - Procoracoid Foramen**: 0. Present; 1. Absent.

**176 - Coracoid**: 0. Large, with posterior process; 1. Small, without posterior process.

**177 - Manubrium Size Relative to Succeeding Sternebrae**: 0. Large; 1. Small.

**178 - Orientation of Glenoid Relative to Plane or Axis of Scapula**: 0. Nearly parallel to the long axis and facing posterolaterally; 1. Oblique and facing more posteriorly; 2. Perpendicular to the main plane of the scapular plate.

**179 - Shape and Curvature of Glenoid**: 0. Saddle-shaped, oval, and elongated; 1. Uniformly concave and more rounded in outline.

**180 - Medial Surface of Scapula**: 0. Concave; 1. Flat.

**181 - Humeral Head**: 0. Subspherical, weakly inflected; 1. Spherical and strongly inflected.

**182 - Intertubercular Groove Separating Deltopectoral Crest From Lesser Tubercle**: 0. Shallow and broad; 1. Narrow and deep.

**183 - Size of Lesser Tubercle of Humerus**: 0. Wider than the greater tubercle; 1. Subequal to narrower than the greater tubercle.

**184 - Torsion Between Proximal and Distal Ends of Humerus**: 0. Strong (>30); 1. Moderate (30–15); 2. Weak (<15).

**185 - Ventral Extension of Deltopectoral Crest or Position of Deltoid Tuberosity**: 0. Not extending beyond the midpoint of the humeral shaft; 1. Extending ventrally (distally) past the midpoint of the shaft.

**186 - Ulnar Articulation on Distal Humerus**: 0. Bulbous ulnar condyle; 1. Incomplete trochlea with vestigial ulnar condyle in anterior view; 2. Trochlea has extending to the anteroventral side.

**187 - Radial Articulation on Distal Humerus**: 0. Distinct and rounded condyle separated from the ulnar articulation in the anteroventral view of the humerus; 1. Radial articulation forms a rounded condyle anteriorly but its posterior surface is nearly cylindrical; 2. Capitulum, radial articulating structure forms a continuous synovial surface with the ulnar trochlea.

**188 - Entepicondyle and Ectepicondyle of Humerus**: 0. Robust; 1. Weak.

**189 - Rectangular Shelf for Supinator Ridge Extended from Ectepicondyle**: 0. Absent; 1. Present.

**190 - Styloid Process of Radius**: 0. Weak; 1. Strong.

**191 - Enlargement of Scaphoid with a Distomedial Projection**: 0. Absent; 1. Present.

**192 - Size and Shape of Hamate (Unciform) in Wrist**: 0. Anteroposteriorly compressed (wider than longer in dorsal view); 1. Mediolaterally compressed (longer than wide).

**193 - Acetabular Dorsal Emargination**: 0. Emarginated; 1. With a complete rim.

**194 - Sutures of Ilium, Ischium, and Pubis Within Acetabulum in Adults**: 0. Unfused; 1. Fused.

**195 - Ischiatic Tuberosity**: 0. Dorsal margin with a small or absent ischiatic tuberosity; 1. Dorsal margin concave and ischiatic tubercle hypertrophied.

**196 - Head of Femur Is Inflected and Set off From Shaft by a Neck**: 0. Neck absent, head oriented dorsally; 1. Neck present and head inflected medially.

**197 - Fovea for Acetabular Ligament on Femoral Head**: 0. Absent; 1. Present.

**198 - Greater Trochanter**: 0. Directed dorsolaterally; 1. Directed dorsally.

**199 - Orientation of Lesser Trochanter**: 0. On the medial side of the shaft; 1. On the ventromedial or ventral side of the shaft.

**200 - Size of Lesser Trochanter**: 0. Large; 1. Small.

**201 - Patellar Groove of Femur**: 0. Absent; 1. Shallow and weakly developed; 2. Well developed.

**202 - Proximolateral Tubercle or Tuberosity of Tibia**: 0. Large and hook-like; 1. Indistinct.

**203 - Distal Tibial Malleolus**: 0. Weak; 1. Distinct.

**204 - Fibula Contacting Distal End of Femur**: 0. Present; 1. Absent.

**205 - Distal Fibular Styloid Process**: 0. Weak or absent; 1. Distinct.

**206 - Fibula Contacting the Calcaneus**: 0. Extensive contact; 1. Reduced; 2. Mortise and tenon contact of fibula to the ankle.

**207 - Superposition of Astragalus Over Calcaneus**: 0. Little or absent; 1. Weakly developed; 2. Present.

**208 - Orientation of Sustentacular Facet of Calcaneus with Regard to Horizontal Plane of Astragalus**: 0. Nearly vertical; 1. Oblique (<70) to nearly horizontal.

**209 - Astragalar Neck**: 0. Absent; 1. Weakly developed; 2. Present.

**210 - Astragalar Trochlea**: 0. Absent; 1. Present.

**211 - Calcaneal Tubercle**: 0. Short, without terminal swelling; 1. Elongated with distal swelling.

**212 - Peroneal Process and Groove of Calcaneus**: 0. Forming laterally directed shelf, and without a distinct process; 1. Weakly developed with shallow groove on the lateral side of process; 2. With a distinct peroneal process.

**213 - Contact of the Cuboid on Calcaneus**: 0. On the anterior end of the calcaneus, the cuboid is aligned with the long axis of the calcaneous; 1. On the anteromedial aspect of the calcaneous, the cuboid is skewed to the medial side of the long axis of the calcaneous.

**214 - Relationships of Proximal End of Metatarsal V to Cuboid**: 0. Metatarsal V is offset from the cuboid; 1. Metatarsal V is far offset from the cuboid, so that it contacts the calcaneous; 2. Metatarsal V is aligned with the cuboid.

**215 - Angle of Metatarsal III to Calcaneus**: 0. Metatarsal III is aligned with (or parallel to) an imaginary line through the long axis of the calcaneous; 1. Metatarsal III is oriented oblique to an imaginary line through the long axis of the calcaneous.

**216 - Sesamoid Bones in Flexor Tendons**: 0. Absent; 1. Present and unpaired; 2. Present and paired.

**217 - Tarsal Spur**: 0. Absent; 1. Present.

**218 - Sharp Constriction of Rostrum in Front of Molariform-Premolar Boundary**: 0. Present; 1. Absent.

**219 - External Size of Cranial Moiety of Squamosal**: 0. Narrow; 1. Broad.

**220 - Participation of Cranial Moiety of Squamosal in Braincase**: 0. Does not participate in the endocranial wall of the braincase; 1. Participates in the endocranial wall of the braincase.

**221 - Neck Between Glenoid and Cranial Moiety of Squamosal**: 0. Absent; 1. Present.

**222 - External Auditory Meatus**: 0. Present as the postcraniomandibular joint sulcus; 1. Absent; 2. Present as a groove on the squamosal, or a notch.

**223 - Position of Craniomandibular Joint**: 0. Posterior or lateral to the level to the fenestra vestibuli; 1. Anterior to the level of the fenestra vestibuli.

**224 - Orientation of Glenoid Fossa**: 0. On the inner side of the zygoma and facing ventromedially; 1. On the platform of the zygoma and facing ventrally.

**225 - Postglenoid Process**: 0. Absent; 1. Present as a distinctive process.

**226 - Postglenoid Foramen Within Squamosal Bone**: 0. Absent; 1. Present.

**227 - Basisphenoid Wing on Ventral Aspect of Skull**: 0. Present, overlapping part of or the whole cochlear housing; 1. Absent.

**228 - Relationship of Pars Cochlearis to Lateral Lappet of Basioccipital**: 0. Pars cochlearis is entirely covered by basioccipital; 1. Pars cochlearis partially covered by basioccipital; 2. Pars cochlearis fully exposed as promontorium.

**229 - Medial Flat Facet of Promontorium of Pars Cochlearis**: 0. Flat; 1. Inflated and convex.

**230 - Ventral Outline and Morphology of Promontorium**: 0. Triangular, with steep and slightly concave lateral wall; 1. Elongated and cylindrical petrosal cochlear housing; 2. Bulbous and oval-shaped promontorium.

**231 - Cochlea**: 0. Short and uncoiled; 1. Elongated and partly coiled; 2. Elongate and coiled to about 360° or more.

**232 - Morphology of Internal Acoustic Meatus**: 0. The floor is ossified and the meatus is a deep tube; 1. Present as a shallow depression; 2. Present and the floor is developed as the cribriform foramina for auditory nerve.

**233 - Primary Bony Lamina Within Cochlear Canal**: 0. Absent; 1. Present.

**234 - Secondary Bony Lamina for Basilar Membrane Within Cochlear Canal**: 0. Absent; 1. Present.

**235 - Crista Interfenestralis**: 0. Horizontal and extending to base of the paroccipital process; 1. Vertical, delimiting the back of the promontorium.

**236 - Postpromontorial Tympanic Recess**: 0. Absent; 1. Present.

**237 - Caudal Tympanic Process of Petrosal**: 0. Absent; 1. Present as a continuous crest; 2. Caudal tympanic process notched.

**238 - Prootic Canal**: 0. Present; 1. Absent.

**239 - Prootic Canal Confluence With Pterygoparoccipital Foramen**: 0. Prootic canal present, and its tympanic aperture is a distinct; separate foramen 1. Prootic canal present, and its tympanic aperture is confluent; with the pterygoparoccipital foramen

**240 - Lateral Trough Floor Anterior to Tympanic Aperture of Prootic Canal and/or Primary Facial Foramen**: 0. Open lateral trough but no bony floor; 1. Present as a bony shelf; 2. Lateral trough absent.

**241 - Enclosure of Geniculate Ganglion by Bony Floor of Petrosal**: 0. Absent; 1. Present.

**242 - Anteroventral Opening of Cavum Epiptericum**: 0. Fully open ventrally; 1. Partially enclosed by petrosal or lateral flange; 2. Enclosed by both the alisphenoid and the petrosal

**243 - Anterior Lamina of Petrosal and Ascending Process of Alisphenoid and Their Relationships to Exit of Mandibular Branch (V3) of Trigeminal Nerve**: 0. V3 foramen placed at the suture of the alisphenoid ascending process and the anterior lamina of petrosal; 1. V3 placed within the enlarged anterior lamina of the petrosal; 2. Double trigeminal foramina within the anterior lamina in addition to the trigeminal foramen at the anterior lamina border with alisphenoid; 3. V3 within the ascending process of the alisphenoid.

**244 - Quadrate Ramus of Alisphenoid**: 0. Forming a rod overlapping with the anterior part of the lateral flange; 1. Present but not extending back too far, mostly laminar process in the vicinity of the oval foramen; 2. Absent.

**245 - Orientation of Anterior Part of Lateral Flange**: 0. Horizontal shelf; 1. Ventrally directed; 2. Vestigial or absent.

**246 - Vascular Foramen in Posterior Part of Lateral Flange Anterior to Pterygoparoccipital Foramen**: 0. Present; 1. Absent.

**247 - Relationship of Petrosal Lateral Flange to Crista Parotica**: 0. Widely separated; 1. Narrowly separated; 2. Continuous bone formed by petrosal.

**248 - Morphology of Pterygoparoccipital Foramen. That is, ramus superior foramen**: 0. Laterally open notch; 1. Foramen enclosed by the petrosal or squamosal or both.

**249 - Position of Pterygoparoccipital Foramen Relative to Fenestra Vestibuli**: 0. Foramen posterior or lateral to the level of the fenestra vestibuli; 1. Foramen anterior to the level of the fenestra vestibuli.

**250 - Bifurcation of Paroccipital Process of Petrosal**: 0. Absent; 1. Present.

**251 - Posterior Paroccipital Process of Petrosal**: 0. No ventral projection below the level of its surrounding structures; 1. Projecting below the surrounding structures.

**252 - Morphological Differentiation of Anterior Paroccipital Region**: 0. Anterior paroccipital region is indistinct from surrounding structures; 1. Anterior paroccipital region is bulbous and distinctive from the surrounding structures; 2. Anterior paroccipital region has a distinct crista parotica.

**253 - Epitympanic Recess Lateral to Crista Parotica**: 0. Absent; 1. Present.

**254 - Relationship of Squamosal on Paraoccipital Process of Petrosal**: 0. Squamosal covers the entire paroccipital region; 1. No squamosal cover on anterior paroccipital region; 2. Squamosal covering a part of the paroccipital region, but not on the crista parotica (the squamosal wall and the cristaparotica are separated by the epitympanic recess).

**255 - Medial Process of Squamosal Reaching Toward Foramen Ovale**: 0. Absent; 1. Present.

**256 - Stapedial Artery Sulcus on Pars Cochlearis of Petrosal**: 0. Absent; 1. Present.

**257 - Transpomontorial Sulcus for Internal Carotid Artery on Pars Cochlearis**: 0. Absent; 1. Present.

**258 - Bullar Process of Alisphenoid**: 0. Absent; 1. Present.

**259 - Hypotympanic Recess in Junction of Alisphenoid, Squamosal, and Petrosal**: 0. Absent; 1. Present.

**260 - Separation of Fenestra Cochleae from Jugular Foramen**: 0. Fenestra cochleae and jugular foramen within the same depression; 1. Separate.

**261 - Channel of Perilymphatic Duct**: 0. Open channel and sulcus; 1. Channel partially or fully enclosed.

**262 - Tensor Tympani Fossa**: 0. Indistinct or very shallow; 1. Deep recess on lateral trough anterior to hiatus Fallopii.

**263 - Stapedial Muscle Fossa**: 0. Absent; 1. Present and in alignment with the crista interfenestralis; 2. Present and lateral to the crista interfenestralis.

**264 - Hypoglossal Foramen**: 0. Indistinct, either confluent with the jugular foramen or sharing a depression with the jugular foramen; 1. Separated from the jugular foramen.

**265 - Shape of Incudo-Mallear Contact**: 0. Trochlear surface on the incus; 1. Trough or saddle-shaped contact on the incus; 2. Flat surface.

**266 - Incus (Quadrate) Neck**: 0. Absent; 1. Present.

**267 - Stapedial Process of Incus (Quadrate)**: 0. Absent; 1. Present.

**268 - Dorsal Plate (=Crus Breve) of Incus (Quadrate)**: 0. Broad plate; 1. Pointed triangle; 2. Reduced.

**269 - Incus—Angle of the Crus Breve to Crus Longum**: 0. Alignment or obtuse angle between stapedial process (crus longum) and the dorsal plate (crus breve); 1. Perpendicular; 2. Acute angle between the crus breve and crus longum.

**270 - Primary Suspension of Incus (Quadrate) on Basicranium**: 0. By squamosal and quadratojugal; 1. By squamosal only; 2. By petrosal (either by the preserved direct contact of incus, or by the inference from the presence of a well-defined crista parotica).

**271 - Quadratojugal Notch in Squamosal**: 0. Present as an independent element in adult; 1. Absent.

**272 - Morphology of Stapes**: 0. Columelliform-macroperforate; 1. Columelliform-imperforate (or microperforate); 2. Bicrurate-perforate.

**273 - Bony Secondary Palate**: 0. Ending anterior to the posterior end of the tooth row; 1. Level with the posterior end of the tooth row; 2. Extending posterior to the tooth row.

**274 - Relationship of Maxilla to Subtemporal Margin of Orbit**: 0. Participating in the rounded subtemporal margin of the orbit; 1. Forming a well-defined edge along the subtemporal margin.

**275 - Pterygopalatine Ridge**: 0. Present; 1. Absent.

**276 - Transverse Process of pterygoid**: 0. Present and massive; 1. Present as the hamulus; 2. Greatly reduced or absent.

**277 - Palatal Width Anterior to Basisphenoid**: 0. Very narrow anterior to the basisphenoid; 1. Intermediate width anterior to the basisphenoid; 2. Palatal width is as broad at the basisphenoid as the internal choanae.

**278 - Vault of Nasopharyngeal Passage Near Pterygoid-Basisphenoid Junction**: 0. Roof of the pharynx is V-shaped in transverse section, narrowing toward the basisphenoid; 1. Roof of the pharynx is U-shaped in transverse section.

**279 - Complete Ossification of Orbital Floor**: 0. Absent; 1. Present.

**280 - Pattern of Orbital Mosaic as Exposed Externally**: 0. Alisphenoid contacts the frontal and parietal; 1. Petrosal anterior lamina contacts the orbitosphenoid thereby separating the alisphenoid from the front and the parietal.

**281 - Outline of Facial Part of Lacrimal**: 0. Large, triangular, and pointed anteriorly; 1. Small and rectangular or crescentic; 2. Excluded from the facial (and preorbital) part of the skull.

**282 - Pila Antotica**: 0. Present; 1. Absent (in adult).

**283 - Fronto-Parietal Suture on Alisphenoid:** 0. Dorsal plate of alisphenoid contacting the frontal by the anterior corner; 1. Dorsal plate of alisphenoid has more extensive contact to the frontal (∼50% of its dorsal border).

**284 - Jugal on Zygoma**: 0. Anterior part of the jugal extends on the facial part of the maxilla and forming a part of the anterior orbit; 1. Anterior part of jugal does not reach the facial part of the maxilla and is excluded from the anterior part of the orbit.

**285 - Maximum Vertical Depth of Zygomatic Arch Relative to Length of Skull**: 0. Between 10% and 20%; 1. Between 5% and 7%.

**286 - Posterior Opening of Post-temporal Canal**: 0. At the junction of the petrosal, squamosal, and tabular; 1. Between the petrosal and the squamosal.

**287 - Anterior Ascending Vascular Channel for Arteria Diploëtica Magnain Temporal Region**: 0. Open groove; 1. Partially enclosed in a canal; 2. Completely enclosed in a canal or endocranial.

**288 - Nuchal (Lambdoidal) Crest**: 0. Crest overhanging the concave or straight dorsal part of the occipital plate; 1. Weak crest with convex dorsal part of the occipital plate.

**289 - Sagittal Crest**: 0. Prominently developed; 1. Weakly developed; 2. Absent.

**290 - Tabular Bone**: 0. Present; 1. Absent.

**291 - Shape of Occipital Condyle**: 0. Bulbous; 1. Ovoid; 2. Subcylindrical.

**292 - Occiput Slope**: 0. Occiput slopes posterodorsally, or vertically from the occipital condyles; 1. Occiput slopes anterodorsally from the occipital condyles.

**293 - Foramina on Dorsal Surface of Nasal**: 0. Absent; 1. Present.

**294 - Septomaxilla**: 0. Present and with a ventromedial shelf; 1. Present and without the septomaxillary shelf; 2. Absent.

**295 - Premaxillary Internarial Process**: 0. Present; 1. Absent.

**296 - Facial Part of Premaxilla Borders on Nasal**: 0. Absent; 1. Present.

**297 - Ossified Ethmoidal Cribriform Plate of Nasal Cavity**: 0. Absent; 1. Present.

**298 - Posterior Excavation of Nasal Cavity Into Bony Sphenoid Complex**: 0. Absent; 1. Present, confluent with the nasal cavity; 2. Present and partitioned from the nasal cavity.

**299 - External Bulging of Braincase in Parietal Region**: 0. Absent; 1. Expanded, the parietal part of the cranial vault is wider than the frontal part, but expansion does not extend to the lambdoidal region; 2. Greatly expanded, expansion of cranial vault extends to lambdoidal region.

**300 - Interparietal**: 0. Present as a separate element in adult; 1. Absent.

**301 - Bony Tentorium Septum**: 0. Present; 1. Absent.

**302 - Overall Size of Vermis**: 0. Small; 1. Enlarged.

**303 - Lateral Cerebellar Hemisphere (Excluding Paraflocculus)**: 0. Absent; 1. Present.

**304 - Lateral Extension of Paraflocculus**: 0. Less than 30% of total cerebellar width; 1. More than 30% of the cerebellar width.

**305 - External Division on Endocast Between Olfactory Lobe and Cerebral Hemisphere (Circular Sulcus)**: 0. Absent; 1. Present.

**306 - Anterior Expansion of Cerebral Hemisphere**: 0. Absent; 1. Developed.

**307 - Expansion of Posterior Cerebral Hemisphere**: 0. Absent; 1. Present.

**308 - Interprismatic Matrix**: 0. On all sides, widely separated prisms; 1. Distinct inter-row sheets; 2. Prisms “shoulder to shoulder,” little interprismatic matrix.

**309 - Outer Aprismatic Zone**: 0. Present; 1. Absent

**310 - Lacrimal Foramen Number**: 0. One; 1. Two; 2. None.

**311 - Lacrimal Foramen Position**: 0. Within orbit; 1. On face.

**312 - Preglenoid Process**: 0. Absent; 1. Present.

**313 - Anterior Lamina of Petrosal**: 0. Absent; 1. Present.

**314 - Anterior Lamina of Petrosal Contribution to Braincase Wall**: 0. Large (present); 1. Small (absent).

**315 - Curved Ridge Connecting Caudal Tympanic Process and Crista Interfenestralis**: 0. Absent; 1. Present.

**316 - “Tympanic Process” of Kielan-Jaworowska**: 0. Absent; 1. Present.

**317 - Fenestra Vestibuli**: 0. Round (stapedial ratio <1.6); 1. Oval (stapedial ratio >1.6).

**318 - Presence of labial cuspulids on lower molars**: 0. Absent; 1. Present (#39 of Harper et al., 2019).

**319 - Primary trigon and talonid crests**: 0. Sharp and continuous; 1. Low and intermittent (#40 of Harper et al., 2019).

**320 - Capacity for embrasure shearing during mastication in molar dentition**: 0. Present; 1. None or vestigial (#41 of Harper et al., 2019).

**321 - Metastyle located on discrete projecting lobe in molars**: 0. Present; 1. Metastyle not on discrete lobe (#44 of Harper et al., 2019).

**6. Additional data of phylogenetic analysis results.**

For the analysis the following characters were considered as additive (ordered): 2, 5, 27, 40, 42, 49, 55, 56, 57, 65, 78, 82, 83, 93, 100, 101, 114, 115, 120, 126, 134, 144, 146, 155, 171, 178, 184, 186, 187, 201, 207, 209, 228, 230, 231, 237, 240, 242, 244, 273, 276, 277, 281, 287, 289, 291, 294, and 299 (following Rougier et al., 2011 —in that dataset they have a different order—).

**Figure S5.** Strict consensus tree of 12 MPTs, with Bremer supports.


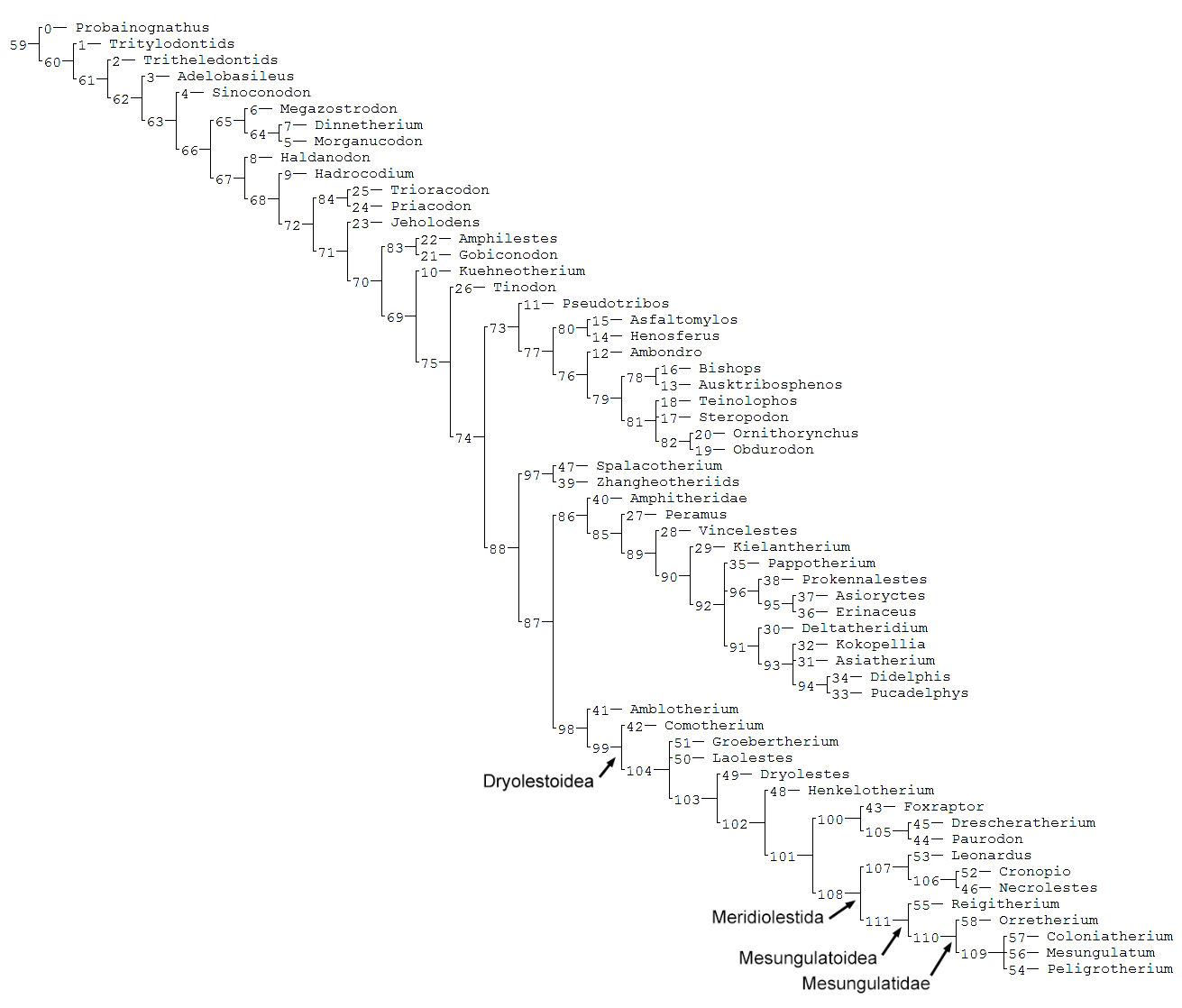


**Figure S6.** Strict consensus tree with node numbers.

**List of synapomorphies common to 12 MPTs**

(Node numbers refer to nodes in consensus tree (Figure S6). Note that characters start from 0)

Probainognathus :

All trees:

No autapomorphies:

Tritylodontids :

All trees:

Char. 6: 0 --> 1

Char. 26: 12 --> 3

Char. 31: 0 --> 1

Char. 32: 0 --> 1

Char. 35: 0 --> 2

Char. 59: 0 --> 4

Char. 103: 0 --> 1

Char. 106: 0 --> 5

Char. 107: 0 --> 1

Char. 109: 1 --> 4

Char. 111: 0 --> 2

Char. 118: 0 --> 2

Char. 134: 0 --> 2

Char. 137: 0 --> 1

Char. 145: 01 --> 3

Char. 242: 0 --> 1

Char. 249: 0 --> 1

Char. 251: 0 --> 1

Char. 253: 0 --> 1

Char. 262: 0 --> 1

Char. 266: 0 --> 1

Tritheledontids :

All trees:

Char. 166: 0 --> 1

Char. 167: 0 --> 1

Char. 169: 0 --> 1

Char. 295: 0 --> 1

Char. 308: 0 --> 1

Char. 310: 0 --> 1

Adelobasileus :

All trees:

Char. 218: 0 --> 1

Char. 247: 0 --> 1

Char. 277: 0 --> 1

Char. 286: 0 --> 1

Char. 290: 1 --> 2

Sinoconodon :

All trees:

Char. 23: 0 --> 1

Char. 26: 1 --> 0

Char. 40: 0 --> 1

Char. 264: 0 --> 1

Char. 290: 1 --> 0

Morganucodon :

All trees:

Char. 26: 1 --> 0

Char. 27: 1 --> 0

Char. 29: 0 --> 2

Char. 140: 0 --> 1

Char. 242: 1 --> 2

Megazostrodon :

All trees:

Char. 44: 0 --> 1

Char. 156: 1 --> 0

Char. 308: 0 --> 1

Dinnetherium :

All trees:

Char. 6: 0 --> 1

Char. 7: 0 --> 1

Char. 23: 0 --> 2

Char. 61: 0 --> 2

Char. 71: 0 --> 1

Char. 75: 2 --> 1

Haldanodon :

All trees:

Char. 23: 0 --> 1

Char. 50: 0 --> 1

Char. 72: 0 --> 2

Char. 105: 0 --> 1

Char. 106: 1 --> 5

Char. 109: 0 --> 3

Char. 119: 0 --> 1

Char. 140: 0 --> 1

Char. 143: 0 --> 1

Char. 154: 1 --> 0

Char. 242: 1 --> 0

Char. 260: 0 --> 1

Char. 284: 1 --> 0

Char. 299: 1 --> 0

Hadrocodium :

All trees:

Char. 8: 0 --> 3

Char. 26: 1 --> 2

Char. 39: 1 --> 0

Char. 48: 0 --> 1

Char. 54: 2 --> 1

Char. 218: 0 --> 1

Char. 220: 1 --> 0

Char. 229: 1 --> 2

Char. 252: 0 --> 1

Char. 287: 0 --> 1

Char. 288: 1 --> 2

Char. 298: 1 --> 2

Kuehneotherium :

All trees:

Char. 2: 1 --> 0

Char. 4: 1 --> 0

Char. 13: 1 --> 0

Char. 16: 2 --> 0

Char. 19: 1 --> 0

Char. 26: 1 --> 0

Char. 39: 2 --> 3

Char. 43: 1 --> 0

Char. 54: 2 --> 3

Char. 61: 1 --> 2

Pseudotribos :

All trees:

Char. 1: 0 --> 123

Char. 16: 2 --> 0

Char. 21: 1 --> 0

Char. 26: 2 --> 0

Char. 49: 0 --> 1

Char. 52: 0 --> 1

Char. 53: 1 --> 0

Char. 72: 1 --> 2

Char. 95: 1 --> 2

Char. 130: 0 --> 1

Char. 146: 0 --> 1

Some trees:

Char. 27: 1 --> 0

Char. 122: 0 --> 1

Ambondro :

All trees:

Char. 43: 1 --> 0

Ausktribosphenos :

Some trees:

Char. 27: 1 --> 0

Henosferus :

All trees:

Char. 21: 1 --> 0

Char. 51: 1 --> 0

Char. 56: 1 --> 2

Asfaltomylos :

All trees:

Char. 64: 0 --> 2

Char. 76: 0 --> 1

Some trees:

Char. 122: 0 --> 1

Bishops :

All trees:

Char. 76: 0 --> 1

Steropodon :

All trees:

No autapomorphies:

Teinolophos :

All trees:

Char. 54: 1 --> 2

Char. 76: 0 --> 1

Obdurodon :

All trees:

Char. 21: 1 --> 0

Char. 154: 01 --> 2

Ornithorynchus :

All trees:

Char. 10: 0 --> 1

Char. 11: 0 --> 1

Char. 108: 1 --> 0

Char. 143: 1 --> 0

Char. 152: 0 --> 1

Char. 153: 0 --> 1

Gobiconodon :

All trees:

Char. 18: 0 --> 1

Char. 27: 1 --> 0

Char. 31: 0 --> 1

Amphilestes :

All trees:

Char. 11: 0 --> 1

Char. 26: 1 --> 2

Char. 35: 1 --> 0

Jeholodens :

All trees:

Char. 27: 1 --> 0

Char. 39: 1 --> 0

Char. 40: 0 --> 1

Char. 44: 1 --> 0

Char. 162: 0 --> 1

Priacodon :

All trees:

Char. 48: 0 --> 1

Trioracodon :

All trees:

Char. 27: 1 --> 0

Char. 39: 1 --> 2

Char. 54: 2 --> 1

Tinodon :

All trees:

Char. 16: 2 --> 1

Char. 26: 2 --> 3

Char. 39: 2 --> 1

Char. 121: 0 --> 1

Char. 132: 1 --> 2

Peramus :

All trees:

Char. 21: 1 --> 0

Char. 45: 0 --> 1

Char. 70: 1 --> 0

Char. 145: 2 --> 1

Vincelestes :

All trees:

Char. 4: 1 --> 2

Char. 31: 0 --> 1

Char. 39: 2 --> 0

Char. 56: 1 --> 2

Char. 61: 02 --> 1

Char. 71: 1 --> 0

Char. 75: 1 --> 0

Char. 83: 1 --> 0

Char. 87: 1 --> 0

Char. 121: 1 --> 0

Char. 127: 1 --> 0

Char. 152: 0 --> 1

Char. 245: 1 --> 0

Char. 272: 2 --> 1

Char. 274: 1 --> 0

Char. 281: 1 --> 0

Char. 288: 1 --> 0

Char. 292: 0 --> 1

Kielantherium :

All trees:

Char. 18: 0 --> 1

Some trees:

Char. 46: 1 --> 0

Deltatheridium :

All trees:

Char. 4: 1 --> 2

Char. 29: 2 --> 1

Char. 61: 0 --> 2

Char. 90: 1 --> 0

Char. 122: 1 --> 0

Char. 132: 0 --> 1

Asiatherium :

All trees:

Char. 298: 1 --> 2

Kokopellia :

All trees:

Char. 18: 0 --> 1

Char. 61: 0 --> 1

Some trees:

Char. 27: 1 --> 0

Char. 33: 1 --> 0

Char. 99: 2 --> 1

Pucadelphys :

All trees:

No autapomorphies:

Didelphis :

All trees:

Char. 1: 3 --> 2

Char. 4: 1 --> 2

Char. 29: 2 --> 1

Char. 40: 0 --> 1

Char. 41: 0 --> 1

Char. 100: 2 --> 0

Char. 127: 1 --> 0

Char. 288: 1 --> 0

Some trees:

Char. 27: 1 --> 0

Pappotherium :

Some trees:

Char. 46: 1 --> 0

Char. 93: 13 --> 2

Erinaceus :

All trees:

Char. 11: 0 --> 1

Char. 31: 0 --> 1

Char. 35: 0 --> 1

Char. 36: 0 --> 1

Char. 39: 2 --> 0

Char. 44: 1 --> 0

Char. 45: 0 --> 1

Char. 64: 0 --> 2

Char. 74: 0 --> 2

Char. 82: 1 --> 2

Char. 85: 4 --> 3

Char. 91: 0 --> 1

Char. 92: 0 --> 1

Char. 94: 0 --> 1

Char. 99: 1 --> 2

Char. 106: 3 --> 5

Char. 109: 2 --> 4

Char. 116: 0 --> 1

Char. 121: 1 --> 0

Char. 127: 1 --> 0

Char. 132: 0 --> 2

Char. 141: 0 --> 1

Char. 142: 0 --> 1

Char. 143: 0 --> 1

Char. 145: 2 --> 3

Char. 298: 1 --> 2

Char. 310: 0 --> 1

Asioryctes :

All trees:

Char. 256: 1 --> 0

Prokennalestes :

All trees:

Char. 18: 0 --> 1

Char. 21: 1 --> 0

Char. 26: 3 --> 2

Char. 39: 2 --> 3

Char. 286: 2 --> 1

Zhangheotheriids :

All trees:

Char. 26: 2 --> 0

Char. 39: 2 --> 1

Char. 40: 0 --> 1

Char. 42: 0 --> 1

Char. 50: 1 --> 0

Char. 56: 1 --> 2

Char. 111: 2 --> 1

Amphitheridae :

All trees:

Char. 51: 1 --> 0

Char. 94: 0 --> 1

Amblotherium :

All trees:

Char. 105: 0 --> 1

Some trees:

Char. 136: 0 --> 1

Comotherium :

All trees:

No autapomorphies:

Foxraptor :

All trees:

Char. 28: 1 --> 0

Char. 43: 0 --> 1

Char. 76: 0 --> 12

Char. 106: 4 --> 5

Char. 108: 1 --> 0

Paurodon :

All trees:

Char. 39: 1 --> 0

Drescheratherium :

All trees:

No autapomorphies:

Necrolestes :

All trees:

Char. 4: 2 --> 1

Char. 41: 1 --> 0

Char. 43: 0 --> 1

Char. 44: 1 --> 0

Char. 56: 1 --> 2

Char. 61: 0 --> 1

Char. 103: 0 --> 1

Char. 126: 2 --> 1

Char. 219: 0 --> 1

Char. 248: 0 --> 1

Char. 294: 1 --> 0

Spalacotherium :

All trees:

Char. 41: 0 --> 1

Char. 74: 0 --> 1

Char. 110: 0 --> 1

Char. 119: 1 --> 2

Char. 138: 1 --> 0

Henkelotherium :

All trees:

Char. 107: 2 --> 0

Char. 125: 1 --> 0

Char. 184: 1 --> 0

Dryolestes :

All trees:

Char. 29: 2 --> 0

Laolestes :

All trees:

Char. 124: 0 --> 1

Some trees:

Char. 105: 0 --> 1

Groebertherium :

All trees:

Char. 72: 0 --> 1

Char. 74: 0 --> 1

Char. 88: 0 --> 1

Char. 121: 1 --> 0

Char. 125: 1 --> 2

Char. 129: 0 --> 1

Char. 142: 0 --> 1

Some trees:

Char. 136: 0 --> 1

Cronopio :

All trees:

Char. 8: 1 --> 3

Char. 21: 1 --> 0

Char. 22: 0 --> 1

Char. 26: 3 --> 1

Char. 29: 2 --> 1

Char. 37: 0 --> 1

Char. 39: 1 --> 2

Char. 53: 1 --> 0

Char. 128: 0 --> 1

Char. 272: 1 --> 0

Char. 276: 1 --> 0

Char. 282: 0 --> 1

Leonardus :

All trees:

Char. 121: 1 --> 0

Peligrotherium :

All trees:

Char. 49: 0 --> 1

Char. 51: 1 --> 2

Char. 95: 0 --> 4

Char. 131: 0 --> 1

Char. 143: 1 --> 2

Char. 236: 1 --> 0

Char. 238: 0 --> 1

Char. 246: 1 --> 0

Character_318_ch_39_de_harper_y_rougier (317): 0 --> 1

Character_319_ch_40_de_harper_and_rougier (318): 0 --> 1

Char. 319: 0 --> 1

Reigitherium :

All trees:

Char. 26: 3 --> 2

Char. 39: 1 --> 2

Char. 67: 1 --> 0

Char. 78: 0 --> 1

Char. 95: 0 --> 4

Char. 113: 1 --> 0

Char. 131: 0 --> 1

Char. 135: 0 --> 1

Character_318_ch_39_de_harper_y_rougier (317): 0 --> 1

Character_319_ch_40_de_harper_and_rougier (318): 0 --> 1

Char. 319: 0 --> 1

Mesungulatum :

All trees:

No autapomorphies:

Coloniatherium :

All trees:

No autapomorphies:

Orretherium :

All trees:

No autapomorphies:

Node 60 :

All trees:

No synapomorphies

Node 61 :

All trees:

Char. 0: 0 --> 1

Char. 24: 0 --> 1

Char. 154: 0 --> 1

Char. 221: 0 --> 1

Char. 269: 0 --> 1

Char. 270: 0 --> 1

Char. 272: 0 --> 1

Char. 290: 0 --> 1

Node 62 :

All trees:

Char. 109: 1 --> 0

Char. 227: 0 --> 1

Char. 239: 0 --> 1

Char. 242: 0 --> 2

Char. 263: 0 --> 1

Char. 276: 0 --> 1

Char. 285: 0 --> 1

Char. 312: 0 --> 1

Node 63 :

All trees:

Char. 68: 0 --> 1

Char. 106: 0 --> 1

Char. 226: 0 --> 1

Char. 240: 0 --> 1

Char. 241: 0 --> 1

Char. 249: 0 --> 1

Char. 259: 0 --> 1

Char. 262: 0 --> 1

Node 64 :

All trees:

Char. 72: 0 --> 1

Char. 228: 0 --> 1

Node 65 :

All trees:

Char. 5: 0 --> 1

Char. 39: 1 --> 3

Char. 51: 0 --> 1

Char. 75: 0 --> 2

Char. 163: 0 --> 1

Char. 196: 0 --> 1

Char. 250: 0 --> 1

Char. 261: 0 --> 1

Node 66 :

All trees:

Char. 27: 0 --> 1

Char. 30: 0 --> 1

Char. 58: 0 --> 1

Char. 59: 0 --> 12

Char. 69: 0 --> 2

Char. 111: 0 --> 1

Char. 170: 0 --> 1

Char. 200: 0 --> 1

Char. 220: 0 --> 1

Char. 227: 1 --> 2

Char. 231: 0 --> 1

Char. 242: 2 --> 1

Char. 253: 0 --> 1

Char. 266: 0 --> 1

Char. 269: 1 --> 2

Char. 293: 0 --> 1

Node 67 :

All trees:

Char. 6: 0 --> 1

Char. 29: 0 --> 2

Char. 184: 0 --> 1

Char. 221: 1 --> 2

Char. 272: 1 --> 2

Char. 274: 0 --> 1

Char. 275: 1 --> 2

Char. 276: 1 --> 2

Char. 277: 0 --> 1

Char. 286: 0 --> 1

Char. 291: 0 --> 1

Char. 304: 0 --> 1

Node 68 :

All trees:

Char. 2: 0 --> 1

Char. 4: 0 --> 1

Char. 44: 0 --> 1

Char. 228: 0 --> 1

Char. 243: 0 --> 1

Char. 281: 0 --> 1

Char. 290: 1 --> 2

Char. 298: 0 --> 1

Char. 305: 0 --> 1

Node 69 :

All trees:

Char. 55: 0 --> 1

Char. 56: 0 --> 12

Char. 66: 0 --> 1

Char. 72: 0 --> 1

Char. 73: 0 --> 2

Char. 77: 0 --> 1

Char. 94: 2 --> 1

Char. 106: 1 --> 2

Char. 109: 0 --> 1

Char. 113: 0 --> 1

Char. 119: 0 --> 1

Char. 140: 0 --> 1

Node 70 :

All trees:

Char. 23: 2 --> 1

Char. 39: 1 --> 2

Char. 67: 0 --> 1

Char. 216: 0 --> 1

Node 71 :

All trees:

Char. 35: 0 --> 1

Char. 112: 0 --> 1

Char. 138: 0 --> 1

Node 72 :

All trees:

Char. 13: 0 --> 1

Char. 14: 0 --> 1

Char. 16: 0 --> 2

Char. 19: 0 --> 1

Char. 22: 0 --> 1

Char. 23: 0 --> 2

Char. 29: 2 --> 1

Char. 61: 0 --> 1

Char. 245: 0 --> 1

Char. 250: 0 --> 1

Char. 262: 1 --> 2

Node 73 :

All trees:

Char. 13: 1 --> 0

Char. 39: 2 --> 3

Char. 86: 0 --> 1

Char. 92: 0 --> 2

Char. 168: 1 --> 0

Char. 170: 2 --> 0

Char. 171: 1 --> 0

Char. 173: 1 --> 0

Char. 177: 1 --> 0

Char. 178: 1 --> 0

Char. 179: 1 --> 0

Char. 185: 1 --> 0

Char. 194: 0 --> 1

Node 74 :

All trees:

Char. 50: 0 --> 1

Char. 55: 1 --> 2

Char. 94: 1 --> 0

Char. 109: 1 --> 2

Char. 111: 1 --> 2

Char. 118: 0 --> 1

Node 75 :

All trees:

Char. 25: 0 --> 1

Char. 26: 1 --> 2

Char. 28: 0 --> 1

Char. 44: 1 --> 0

Char. 68: 1 --> 0

Node 76 :

All trees:

Char. 46: 1 --> 2

Char. 116: 0 --> 1

Node 77 :

All trees:

Char. 8: 0 --> 2

Char. 54: 2 --> 1

Char. 59: 2 --> 3

Char. 60: 0 --> 1

Char. 62: 0 --> 1

Char. 63: 0 --> 1

Char. 65: 0 --> 2

Char. 69: 1 --> 0

Char. 82: 0 --> 2

Char. 83: 0 --> 1

Char. 84: 0 --> 1

Char. 87: 0 --> 1

Char. 93: 0 --> 1

Char. 115: 0 --> 1

Node 78 :

All trees:

Char. 16: 2 --> 1

Char. 42: 0 --> 1

Char. 45: 0 --> 1

Char. 47: 0 --> 1

Char. 61: 1 --> 0

Char. 64: 1 --> 2

Char. 123: 0 --> 1

Char. 143: 1 --> 0

Some trees:

Char. 122: 0 --> 1

Node 79 :

All trees:

Char. 62: 1 --> 2

Char. 64: 0 --> 1

Char. 71: 1 --> 0

Char. 93: 1 --> 3

Char. 106: 3 --> 5

Char. 113: 1 --> 2

Node 80 :

All trees:

Char. 70: 0 --> 1

Char. 72: 1 --> 0

Char. 73: 2 --> 0

Char. 143: 1 --> 0

Node 81 :

All trees:

Char. 20: 0 --> 1

Char. 73: 2 --> 1

Char. 74: 0 --> 2

Char. 83: 1 --> 2

Char. 84: 1 --> 0

Char. 85: 4 --> 3

Char. 118: 1 --> 0

Char. 119: 1 --> 2

Char. 144: 0 --> 1

Node 82 :

All trees:

Char. 4: 1 --> 2

Char. 78: 0 --> 1

Char. 134: 1 --> 2

Some trees:

Char. 7: 1 --> 0

Char. 13: 0 --> 1

Char. 18: 0 --> 1

Char. 22: 0 --> 1

Char. 29: 2 --> 1

Node 83 :

All trees:

Char. 1: 01 --> 3

Node 84 :

All trees:

Char. 11: 0 --> 1

Char. 18: 0 --> 1

Char. 60: 0 --> 1

Char. 76: 0 --> 1

Char. 103: 0 --> 1

Char. 104: 0 --> 1

Char. 261: 0 --> 1

Node 85 :

All trees:

Char. 1: 0 --> 12

Char. 17: 0 --> 1

Char. 54: 3 --> 12

Char. 106: 4 --> 3

Char. 130: 0 --> 1

Node 86 :

All trees:

Char. 44: 0 --> 1

Char. 59: 2 --> 3

Char. 63: 0 --> 1

Char. 83: 0 --> 1

Char. 85: 0 --> 2

Char. 86: 0 --> 1

Char. 87: 0 --> 1

Char. 93: 0 --> 1

Char. 109: 2 --> 1

Char. 115: 0 --> 1

Char. 143: 1 --> 0

Node 87 :

All trees:

Char. 43: 1 --> 0

Char. 60: 0 --> 1

Char. 61: 1 --> 0

Char. 69: 1 --> 0

Char. 70: 0 --> 1

Char. 72: 1 --> 0

Char. 73: 2 --> 0

Char. 121: 0 --> 1

Char. 145: 1 --> 2

Char. 204: 0 --> 1

Char. 255: 0 --> 1

Char. 256: 0 --> 1

Node 88 :

All trees:

Char. 54: 2 --> 3

Char. 127: 0 --> 1

Char. 167: 0 --> 1

Char. 180: 0 --> 1

Char. 182: 0 --> 1

Char. 186: 0 --> 1

Char. 195: 0 --> 1

Char. 196: 0 --> 1

Char. 197: 0 --> 1

Char. 201: 0 --> 1

Char. 207: 0 --> 1

Char. 210: 0 --> 1

Char. 234: 0 --> 1

Char. 235: 0 --> 1

Char. 236: 0 --> 1

Char. 252: 0 --> 1

Char. 253: 1 --> 2

Char. 283: 1 --> 0

Node 89 :

All trees:

Char. 16: 2 --> 1

Char. 82: 0 --> 1

Char. 84: 0 --> 1

Char. 96: 0 --> 1

Char. 146: 0 --> 1

Some trees:

Char. 60: 1 --> 0

Node 90 :

All trees:

Char. 26: 2 --> 3

Char. 51: 1 --> 0

Char. 65: 0 --> 1

Char. 85: 2 --> 4

Char. 113: 1 --> 2

Char. 117: 0 --> 1

Node 91 :

All trees:

Char. 51: 0 --> 1

Char. 91: 0 --> 1

Some trees:

Char. 1: 2 --> 3

Char. 11: 0 --> 1

Char. 15: 0 --> 1

Char. 39: 2 --> 1

Char. 255: 1 --> 0

Char. 256: 1 --> 0

Char. 258: 0 --> 1

Char. 291: 1 --> 0

Node 92 :

All trees:

Char. 89: 0 --> 1

Char. 90: 0 --> 1

Char. 100: 0 --> 1

Char. 109: 1 --> 2

Char. 114: 1 --> 2

Char. 122: 0 --> 1

Node 93 :

All trees:

Char. 64: 0 --> 2

Char. 74: 0 --> 1

Char. 79: 0 --> 1

Char. 82: 1 --> 2

Char. 100: 1 --> 2

Char. 121: 1 --> 0

Some trees:

Char. 57: 0 --> 1

Char. 99: 0 --> 2

Char. 310: 0 --> 1

Node 94 :

All trees:

Char. 89: 1 --> 2

Char. 101: 0 --> 1

Char. 102: 0 --> 1

Some trees:

Char. 22: 0 --> 1

Char. 192: 0 --> 1

Char. 299: 1 --> 0

Node 95 :

All trees:

Char. 4: 1 --> 2

Char. 100: 1 --> 2

Char. 237: 0 --> 1

Char. 314: 0 --> 1

Char. 315: 0 --> 1

Char. 316: 0 --> 1

Node 96 :

All trees:

Char. 48: 1 --> 0

Char. 52: 0 --> 1

Char. 98: 0 --> 1

Char. 236: 1 --> 2

Some trees:

Char. 57: 0 --> 1

Char. 99: 0 --> 1

Node 97 :

All trees:

Char. 1: 0 --> 2

Char. 5: 1 --> 0

Char. 49: 0 --> 1

Char. 107: 0 --> 1

Char. 128: 0 --> 1

Some trees:

Char. 136: 0 --> 1

Node 98 :

All trees:

Char. 26: 2 --> 3

Char. 27: 1 --> 0

Char. 75: 1 --> 0

Char. 107: 0 --> 2

Char. 110: 0 --> 1

Char. 119: 1 --> 2

Char. 122: 0 --> 1

Char. 135: 0 --> 1

Char. 140: 1 --> 0

Node 99 :

All trees:

Char. 126: 1 --> 2

Node 100 :

All trees:

Char. 0: 1 --> 0

Char. 59: 2 --> 3

Char. 85: 0 --> 2

Char. 92: 0 --> 2

Char. 107: 2 --> 1

Char. 110: 1 --> 0

Node 101 :

All trees:

Char. 27: 0 --> 1

Char. 44: 0 --> 1

Char. 45: 0 --> 1

Char. 54: 3 --> 2

Char. 105: 0 --> 1

Char. 124: 0 --> 1

Char. 128: 1 --> 0

Char. 143: 1 --> 0

Node 102 :

All trees:

Char. 39: 2 --> 1

Char. 135: 1 --> 0

Node 103 :

All trees:

Char. 127: 1 --> 0

Char. 138: 1 --> 0

Node 104 :

All trees:

Char. 125: 0 --> 1

Char. 128: 0 --> 1

Node 105 :

All trees:

Char. 40: 0 --> 1

Node 106 :

All trees:

Char. 105: 1 --> 0

Char. 124: 1 --> 0

Char. 125: 12 --> 0

Char. 133: 1 --> 0

Char. 134: 1 --> 0

Char. 145: 1 --> 0

Node 107 :

All trees:

Char. 51: 1 --> 2

Char. 145: 2 --> 1

Char. 152: 0 --> 1

Char. 153: 0 --> 1

Node 108 :

All trees:

Char. 4: 1 --> 2

Char. 41: 0 --> 1

Char. 54: 2 --> 1

Char. 80: 1 --> 0

Char. 81: 0 --> 1

Char. 129: 0 --> 1

Char. 136: 0 --> 1

Char. 284: 1 --> 0

Char. 320: 0 --> 1

Node 109 :

All trees:

Char. 148: 0 --> 1

Char. 150: 0 --> 1

Node 110 :

All trees:

Char. 42: 0 --> 1

Char. 44: 1 --> 0

Char. 48: 1 --> 2

Char. 70: 1 --> 0

Char. 107: 2 --> 1

Char. 128: 0 --> 1

Char. 141: 0 --> 1

Char. 142: 0 --> 1

Char. 143: 0 --> 1

Char. 149: 0 --> 1

Node 111 :

All trees:

Char. 41: 1 --> 2

Char. 43: 0 --> 1

Char. 72: 0 --> 2

Char. 74: 0 --> 2

Char. 106: 4 --> 5

**7. Bibliography for Supplementary Data**

Bonaparte, J. F. Sobre *Mesungulatum houssayi* y nuevos mamíferos cretácicos de Patagonia. 4° Congreso Argentino de Paleontología y Bioestratigrafía, Mendoza, Actas 2:48–61 (1986).

Bonaparte, J. F. New Late Cretaceous mammals from the Los Alamitos Formation, northern Patagonia. National Geographic Research 6:63–93 (1990).

Harper, T., Parras, A. & Rougier, G. W. *Reigitherium* (Meridiolestida, Mesungulatoidea) an enigmatic Late Cretaceous mammal from Patagonia, Argentina: morphology, affinities, and dental evolution. J Mammal Evol Evol 26:447–478 (2019).

Páez Arango, N. Dental and craniomandibular anatomy of *Peligrotherium tropicalis*: the evolutionary radiation of South American dryolestoid mammals. Unpublished Ms. Thesis, University of Louisville, 107 p. (2008).

Rougier, G. W., Forasiepi, A. M., Hill, R. V. & Novacek, M. J. New mammalian remains from the Late Cretaceous La Colonia Formation, Patagonia, Argentina. ‎Acta Palaeontol Pol 54:195–212 (2009).

Rougier, G. W., Apesteguía, S. & Gaetano, L. C. Highly specialized mammalian skulls from the Late Cretaceous of South America. Nature 479: 98–102 (2011).

Rougier, G. W., Wible, J. R., Beck, R. M. D. & Apesteguía, S. The Miocene mammal *Necrolestes* demonstrates the survival of a Mesozoic nontherian lineage into the late Cenozoic of South America. Proc Natl Acad Sci USA 109:20053–20058 (2012).

Rougier, G. W., Martinelli, A. G. & Forasiepi, A. M. Mesozoic Mammals from South America and Their Forerunners. . Springer Earth System Sciences, 10.1007/978-3-030-63862-7 (2021).

Wible, J. R. & Rougier, G. W. Craniomandibular anatomy of the subterranean meridolestidan *Necrolestes patagonensis* Ameghino, 1891 (Mammalia, Cladotheria) from the Early Miocene of Patagonia. Ann Carnegie Mus 84:183–251 (2017).
